# Identification of Cathepsin H and Metabolic Traits as Potential Biomarkers for Lung Cancer by Mendelian Randomization and Single‐Cell Transcriptomics

**DOI:** 10.1002/ggn2.202500012

**Published:** 2025-11-14

**Authors:** Chenghu Song, Weici Liu, Zhao He, Jiwei Liu, Ruixin Wang, Lei Wu, Yize Wang, Mingfeng Zheng, Dong Tian, Wenjun Mao

**Affiliations:** ^1^ Department of Thoracic Surgery The Affiliated Wuxi People's Hospital of Nanjing Medical University, Wuxi People's Hospital Wuxi Medical Center Nanjing Medical University Wuxi China; ^2^ Department of Thoracic Surgery West China Hospital Sichuan University Chengdu China

**Keywords:** capthesin H, lung adenocarcinoma, metabolic traits, Mendelian Randomization, single‐cell transcriptome

## Abstract

Lung cancer is a major global malignancy with debated roles for cathepsin H (CTSH), a lysosomal protease, and underexplored regulation by metabolites. We analyzed lung cancer incidence and hyperglycemia‐related mortality trends (1990‐2021) using Joinpoint regression. Mendelian randomization (MR), meta‐analysis, and two‐step mediation examined CTSH and 233 metabolic traits. Single‐cell RNA sequencing (scRNA‐seq) and TCGA/HPA datasets validated CTSH expression. Lung cancer incidence decreased overall but rose in women, while fasting hyperglycemia‐related mortality increased. CTSH elevated lung cancer and adenocarcinoma risks, with docosahexaenoic acid (22:6) and omega‐3 fatty acids driving adenocarcinoma progression. A higher linoleic acid (18:2)/total fatty acid ratio reduced risk. scRNA‐seq identified CTSH in myeloid cells, especially “mo‐Mac,” which promoted tumors. CTSH expression patterns were evaluated using TCGA and HPA data, revealing protein‐level overexpression in tumors with some divergence from transcriptomic results. CTSH is linked to lung cancer, particularly adenocarcinoma, with modest effects mediated by metabolites like omega‐3 fatty acids. Its prominent expression in macrophages suggests novel therapeutic targets. These findings, though consistent, require further validation due to modest effect sizes and dataset heterogeneity.

## Graphical abstract

This study explores the link between CTSH and lung cancer, covering global trends in lung cancer and hyperglycemia, MR analysis of CTSH and 233 metabolic traits, and scRNA analysis, along with multi‐level validation of CTSH expression in LUAD. These findings offer valuable insights into lung cancer biology and provide new perspectives for biomarker discovery and targeted therapies.

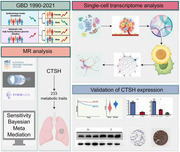

**Abbreviations**
APC: Annual percent change
AAPC: Average annual percent change
BWMR: Bayesian weighted Mendelian randomization
CI: Confidence intervals
CTSH: Cathepsin H
DHA: Docosahexaenoic acid
FDR: False discovery rate
GWAS: Genome‐wide association study
GBD: Global Burden of Disease
HPA: Human protein atlas
IVs: Instrumental variables
IVW: Inverse variance weighting
LUAD: Lung Adenocarcinoma
LUSC: Lung Squamous Cell Carcinoma
MR: Mendelian randomization
PCA: Principal component analysis
PPI: Protein protein interaction
SCLC: Small Cell Lung Cancer
SNPs: Single nucleotide polymorphisms
scRNA: Single‐cell RNA
TCGA: The Cancer Genome Atlas
UMAP: Uniform Manifold Approximation and Projection
WB: Western Blot

## 1 Introduction

Lung cancer was one of the most common and deadly malignancies worldwide. Despite significant advances in early diagnosis, precision treatment, and immunotherapy in recent years, dacomitinib as a first‐line treatment showed significant efficacy and manageable safety in a multicenter study in China, the overall 5‐year survival rate for lung cancer patients remains low, at approximately 20% Despite advances in early diagnosis, precision treatment, and immunotherapy like dacomitinib that showed significant efficacy and safety as a first‐line therapy for EGFR 21L858R‐mutated NSCLC in a Chinese multicenter study [1], the 5‐year survival rate for lung cancer remains low at ∼20% [2, 3]. The pathogenesis of lung cancer was complicated, involving interactions among genetic susceptibility, environmental factors, epigenetic regulation, and the immune microenvironment [4, 5]. However, many critical molecular mechanisms remained unknown [6, 7].

It was well known that many studies have reported a close relationship between hyperglycemia and lung cancer [8, 9]. Recent studies have found that a high‐glucose environment could increase the levels of cathepsins [10, 11]. Cathepsin H (CTSH), as a member of the cysteine protease family, had also attracted attention for its potential role in the pathogenesis of lung cancer [12, 13]. Previous studies had shown that CTSH was abnormally expressed in various cancers (including breast, prostate, and colorectal cancers) and was involved in processes such as tumor invasion, metastasis, angiogenesis, and drug resistance [14, 15]. Gocheva et al. discovered that CTSH promoted cancer metastasis primarily through the degradation of extracellular matrix proteins [16]. Although a previous Mendelian randomization (MR) study had preliminarily established a causal link between lung cancer and CTSH [17], single database‐derived results might be coincidental due to sample population differences. Heterogeneity among populations can lead to biased outcomes, diminishing the findings' credibility and scientific validity. Notably, CTSH has also been found to be involved in cellular metabolic regulation, including amino acid, lipid, and glucose metabolism. For example, alterations in amino acid or glucose utilization can enhance tumor cell survival [18], while reprogramming of lipid metabolism plays a crucial role in the progression of lung cancer [19]. Thus, broader research is required to elucidate CTSH's role in lung cancer.

MR used genetic variation from genome‐wide association studies (GWAS) as an instrumental variable to determine causal relationships between exposures and outcomes [20]. Simultaneously, MR analysis addressed the limitations of previous observational studies. To mitigate biases from single‐population studies, our study employed a meta‐analysis, synthesizing GWAS data on lung cancer and its subtypes from diverse sources. This elevated the evidence quality and enhanced credibility. We then used a two‐step method to explore the mediating effects of 233 metabolic traits, deepening our understanding of CTSH's role in lung cancer. Finally, Bayesian colocalization analysis further corroborated the causal relationship. While MR excelled in inferring causality, it lacked the ability to explain underlying mechanisms. The advances in single‐cell RNA (scRNA) technology have led to more precise studies of the underlying mechanisms. Herein, we analyzed global lung cancer incidence trends from 1990 to 2021 and mortality rates attributable to fasting hyperglycemia. To address the limitations of previous studies, we integrated multiple databases to validate the relationship between CTSH and lung cancer, and also conducted an in‐depth analysis of the mediating effects of 233 metabolic traits. Ultimately, scRNA analysis was performed to elucidate the strong correlation between CTSH and myeloid cells, revealing differences in RNA and protein expression.

## 2 Materials and Methods

### 2.1 Study Design

**FIGURE 1:**
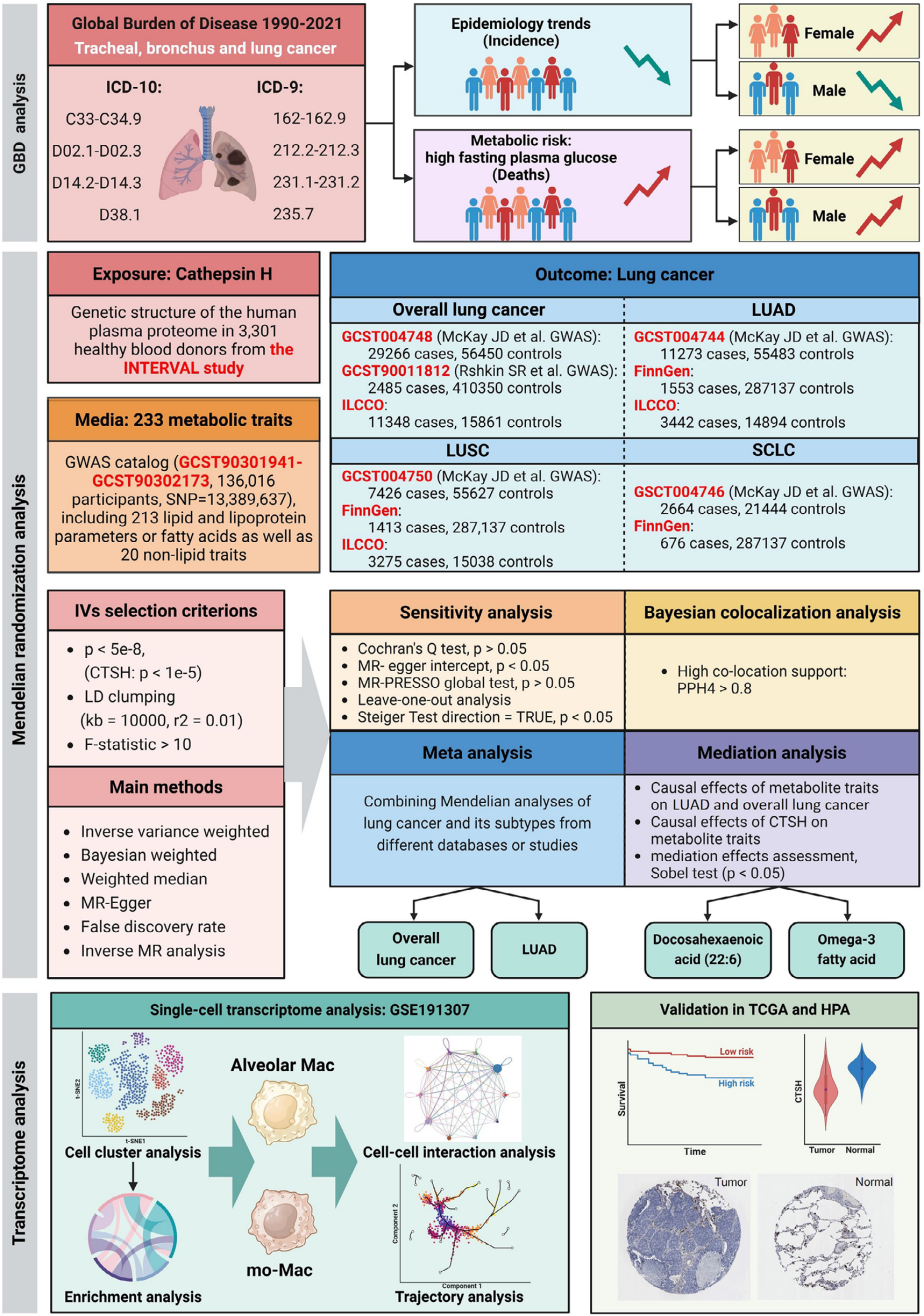
Overall flow of the research. Created with BioRender.com.

The study design is outlined in Figure 1. First, we used Global Burden of Disease (GBD) 2021 data to perform regression analyses on global lung cancer incidence and mortality rates due to fasting hyperglycemia from 1990 to 2021. We then employed the MR method to analyze the risk relationship between CTSH and lung cancer subtypes from multiple databases, conducted a meta‐analysis, and identified subtypes closely related to CTSH. Subsequently, we performed MR analysis on 233 metabolic traits and the identified lung cancer subtypes, evaluating the causal association and calculating the mediation proportion of each metabolite. Finally, we analyzed CTSH expression patterns in single‐cell types using the GEO database and validated CTSH expression in normal and tumor groups using The Cancer Genome Atlas (TCGA) and the Human Protein Atlas (HPA) databases. To avoid sample overlap, exposure and outcome data were sourced from different databases, and all data were publicly available, thus not requiring further ethical approval.

### 2.2 Data Sources

Data on global lung cancer incidence and mortality rates linked to fasting hyperglycemia were sourced from the Global Health Data Exchange, encompassing 204 countries and regions, and 811 areas. The diagnostic criteria for lung cancer met the International Classification of Diseases, 9th and 10th editions (ICD‐10: C33‐C34.9, D02.1‐D02.3, D14.2‐D14.3, D38.1; ICD‐9: 162‐162.9, 212.2‐212.3, 231.1‐231.2, 235.7).

The genetic instrument for assessing CTSH levels (µg/L) came from the INTERVAL study, which included 3,301 Europeans [21]. The GWAS summary data for lung cancer were also from European populations. One GWAS study by Rshkin SR et al. included 2485 lung cancer patients and 410 350 controls [22]. Another GWAS summary data by McKay JD et al. included 29 863 lung cancer patients and 55 586 controls [23], including 11 273 adenocarcinomas, 7,426 squamous cell carcinomas, and 2,664 small cell lung cancers. Furthermore, the FinnGen database currently lacks GWAS summary data on overall lung cancer [24]. Consequently, our analysis focused on the available data, which includes 1,553 cases of lung adenocarcinoma, 1,413 cases of squamous cell carcinoma, 676 cases of small cell lung carcinoma, and 287 137 control subjects. Another study from the ILCCO database [25] included 11 348 lung cancer patients and 15 861 controls, including 3,442 adenocarcinomas and 3,275 squamous cell carcinomas. Mediator studies utilized data on 233 circulating metabolites, including 213 lipids and 20 non‐lipid traits, from a recent genome‐wide association study that analyzed 33 cohorts, 13 389 637 SNPs, and 136 016 participants [26].

Bulk RNA‐sequencing data for lung adenocarcinoma were downloaded from TCGA to analyze CTSH differential expression and prognostic performance between tumor and normal groups. Pathological and normal tissue staining images were sourced from the HPA database. The lung cancer scRNA‐seq dataset was obtained from GSE131907 and processed using Seurat (v5.1.0). The TCGA‐LUAD dataset (*n* = 477 tumor samples, 59 normal samples, log2‐normalized RSEM processing) was used for CTSH differential expression and survival analysis, with the tumor group defined as confirmed lung adenocarcinoma samples and the normal group as adjacent tissues. The HPA dataset was used for protein expression validation, with the tumor group defined by pathological diagnosis.

### 2.3 Joinpoint Regression Model

This study utilized the Joinpoint 4.9.1.0 software from the National Cancer Institute to perform a multi‐segment regression analysis on the time trends of lung cancer incidence and mortality attributable to fasting hyperglycemia [27]. The main metrics utilized in the study included the annual percent change (APC), average annual percent change (AAPC), and 95% confidence intervals (CI). APC or AAPC greater than 0 indicated an increasing trend in lung cancer during that period, whereas values less than 0 indicated a decreasing trend. Statistical significance was set at *p* < 0.05.

### 2.4 MR Analysis

In our study, the inverse variance weighted (IVW) method [28], known for its robustness, was chosen as the primary method for evaluating causality. We also performed false discovery rate (FDR) correction and combined estimates from different sources using meta‐analysis methods [29]. Additional methods, such as MR‐Egger [30] and weighted median [31], were used to confirm the reliability of the MR findings. Additionally, we used the weighted mode and simple mode methods [32], with the former assigning weights to each SNP and the latter using the effect estimate of the single instrumental variable when only one was available. Notably, we adopted the latest Bayesian weighted Mendelian randomization (BWMR) method [33], which avoids the issue of pleiotropy violating the assumptions of instrumental variables, allowing for causal inference even in the presence of pleiotropy. Post‐MR analysis, the Steiger Direction Test was utilized to discern and preclude reverse causality between the exposure and outcome variables [34]. Additionally, we performed sensitivity analyses to assess the robustness of our results, including MR pleiotropy residual sum and outlier (MR‐PRESSO), Egger intercept, Cochran's Q, leave‐one‐SNP, and leave‐one‐dataset analysis. Furthermore, the “product of coefficients” methodology was applied to quantify the indirect impact of CSTH on the risk of lung cancer subtypes through potential mediating factors. The delta method was used to determine the standard error of the indirect effect. Finally, we further performed an inverse MR analysis for all results with significance.

In genetic instrumental variable selection, we have included a compact SNP table to perform clumping to ensure SNP independence (r^2^< 0.001, 10 000 kb). Harmonization ensured consistent effect alleles across datasets by comparing effect directions and allele frequencies, excluding inconsistent SNPs (Table S11). These steps were implemented using the “TwoSampleMR” package in R. Cochran's Q test was utilized to assess SNP heterogeneity (*p* > 0.05). When significant heterogeneity among SNPs was detected, a random effects model was used; otherwise, a fixed effects model was applied [35]. The MR‐PRESSO global test and MR‐Egger intercept were employed to detect outliers and assess the presence of horizontal pleiotropy [36]. The MR‐Egger intercept serves as a measure of average pleiotropy. When significant horizontal pleiotropy was detected, the MR‐PRESSO outlier test was utilized to address this issue by adjusting or removing the outliers. We also did a leave‐one‐out analysis to find SNPs that could greatly impact the estimates [37].

### 2.5 Bayesian Colocalization Analysis

Bayesian colocalization analysis was employed to assess whether two traits share a common variant within a specific chromosomal region. This method considered all SNPs in the region and provided valuable insights into the genetic factors influencing both traits, which MR analysis did not address [38]. Posterior probabilities were computed for each hypothesis (H0, H1, H2, H3, and H4), and the analysis was carried out with default parameters. A shared causal variant (PH4) was deemed robust if its posterior probability was ≥0.8, indicating colocalization of the two traits in the specified region.

### 2.6 Cellular Constitution Extraction, Re‐clustering, and Enrichment Analysis

The target gene‐enriched cell clusters were visualized in the initial Seurat object. Differences in cell clusters between the Tumor and Normal groups and the number of cells in each cluster were visualized. The subset function was utilized to extract the target cell clusters, which underwent repeated application of dimensionality reduction and annotation processes. Differentially significant cells were identified, and highly variable genes were extracted utilizing the “FindAllMarkers” function for functional enrichment analysis through Gene Ontology (GO) and the Kyoto Encyclopedia of Genes and Genomes (KEGG) by virtue of the R package clusterProfiler and org.Hs.eg.db. We further supplemented the Gene Set Enrichment Analysis (GSEA), filtering up to the top 5 enriched pathways based on the criterion of *P* < 0.05 and NES in descending order, followed by the generation of pathway enrichment plots.

### 2.7 Cell–Cell Interaction Analysis

We used the R package CellChat (version 1.6.1) to project the interactions onto the feature cell subgroups, identifying cell‐cell interactions. Secreted Signaling was selected as the analysis database. The cut‐off standard was set at 25% with min.cell = 10, following the default recommendation of the CellChat package to filter low‐quality signals. These default parameters are widely applied across CellChat analyses to ensure stable communication inference. While alternative thresholds were not retested in this study, we acknowledge this as a limitation and expect the overall interaction trends to remain robust.

### 2.8 Trajectory Analysis

All myeloid cells, along with the selected Alveolar Mac and mo‐Mac subgroups, formed the new input data for the study using the Monocle2 algorithm (R Package Version 2.30.1). This approach provided insights into the dynamic biological processes, such as mutual transformation and evolutionary trajectories of these two cell types.

### 2.9 RNA Extraction and RT‐qPCR

Total RNA was extracted from both cell lines and clinical tissue specimens using TRIzol reagent (Invitrogen; Thermo Fisher Scientific, Inc.), followed by cDNA synthesis using the PrimeScript Reverse Transcription Kit (Takara Bio, Inc.). For quantification of CTSH expression, RT‐qPCR was performed with specific primers: CTSH primer: forward 5'‐TGCCTTTGAGGTGACTCAGG‐3'and reverse 5'‐GCGCTCGATGAGGAAGTACC‐3'. Relative gene expression was normalized to GAPDH and calculated using the 2‐ΔΔCT method.

### 2.10 Western Blotting (WB)

Total protein was extracted from the clinical tissue specimens using RIPA lysis buffer (Meilun Biotech, China) and quantified using the bicinchoninic acid protein assay (Thermo Fisher Scientific). Equal protein amounts were subjected to SDS‐PAGE and transferred onto PVDF membranes (EMD Millipore Corp). Membranes were blocked with 5% BSA for 2 h at room temperature and incubated overnight at 4°C with the primary antibody against CTSH (Proteintech, 1:1000) and β‐actin (Proteintech, 1:1000). After washing three times with TBST, the membranes were incubated with the horseradish peroxidase‐labeled secondary antibody (1:10000; Jackson ImmunoResearch) for 2 h. The protein bands were visualized using enhanced chemiluminescence (Millipore) and captured with the Tanon 4600 imaging system (Tanon Science, China). β‐actin was utilized to normalize the target protein expression.

### 2.11 Statistical Analysis

MR used genetic variations as instrumental variables (IVs) to assess the causal effect of an exposure on an outcome. Effective IVs must meet three criteria: they should be strongly associated with the exposure, not linked to confounders, and influence the outcome only through the exposure. If these conditions are violated, the SNP shows horizontal pleiotropy. For CTSH, due to the limited number of genomes‑wide significant SNPs, a threshold of *p* < 5 × 10^−6^ was adopted for selecting instruments, following prior MR studies on protein traits with constrained instrument availability. For metabolites, where sufficient SNPs are typically available, we applied the conventional genome‑wide significance threshold of *p* < 5 × 10^−8^. To control type I error, false discovery rate (FDR, *q* < 0.05) corrections were applied separately within each analytical framework, including the MR analyses across 233 metabolites and the mediation tests, thereby ensuring robustness to multiple testing. The following conditions were also met: (a) LD measurements among genetic instruments within 10 000 kb were r^2^ < 0.001. (b) Strong instrumental variables with F > 10 were selected using the formula F = (β_expose/Se_expose)^2^ [39]. (c) SNPs with pleiotropy or heterogeneity *p* < 0.05, were removed.

Data pre‐processing involved log1p normalization using Seurat v5.1.0's NormalizeData function for scRNA‐seq data (GSE131907), with batch effects corrected by IntegrateData and outlier cells (<5%) identified and removed via UMAP visualization. TCGA‐LUAD RNA‐sequencing data were log2‐normalized using RNA‐Seq by Expectation‐Maximization (RSEM). The two datasets are from single sources, so batch effects do not need to be removed. For GWAS data, no additional pre‐processing was required beyond the standard quality control applied in the source databases (removal of low‐quality SNPs based on minor allele frequency and imputation info scores). Outliers in MR analyses were evaluated and removed using the MR‐PRESSO test. Continuous variables are presented as mean ± standard deviation SD or median ± interquartile range (IQR) for non‐normal data, with CTSH expression levels shown as log2(transcripts per million + 1) median ± IQR in violin plots. Categorical variables are presented as frequencies and percentages. Sample sizes (n) for key analyses were as follows: CTSH expression comparison based on 5,452 mo‐Mac cells and 11 824 Alveolar Mac cells from GSE131907; survival analysis based on 477 TCGA‐LUAD samples, where more than 20% of samples with genes having a value of 0 were discarded; MR analyses for CTSH involved 3,301 participants from the INTERVAL study for exposure and varying outcome cohorts (29 863 cases and 55 586 controls from McKay et al. [23]); metabolic trait analyses included 136 016 participants from 33 cohorts [26]; Joinpoint regression used data from 204 countries/regions and 811 areas via the GBD 2021 database.

Furthermore, statistical significance was assessed using two‐sided tests unless otherwise specified, with *α* = 0.05. Differences in CTSH expression were evaluated by the Wilcoxon rank‐sum test, with *p*‐values < 0.05 considered significant; survival curves were analyzed by the Kaplan‐Meier method with the log‐rank test. MR analysis employed the IVW method as primary, validated by MR‐Egger and weighted median methods, assuming instrument variable relevance, exclusivity, and no confounding (violations checked via MR‐Egger intercept for pleiotropy and Steiger test for directionality). Joinpoint regression modeled trends with APC and average APC, assuming piecewise linearity. All analyses assumed data independence, with violations checked via Cochran's Q test for heterogeneity (*p* > 0.05 indicating no heterogeneity; random‐effects model applied if detected). No post‐hoc tests were required, as analyses did not involve multiple group comparisons beyond FDR adjustment for multiple testing in MR frameworks.

All statistical analyses were performed using R version 4.3.1 (https://www.r‐project.org), with two‐sample MR analysis conducted using the “TwoSampleMR” and “ieugwasr” packages. Bayesian colocalization analysis utilized the “coloc” package. Single‐cell analyses were performed with Seurat (v5.1.0), clusterProfiler, org.Hs.eg.db, CellChat (v1.6.1), and Monocle2 (v2.30.1). Joinpoint regression used the Joinpoint 4.9.1.0 software from the National Cancer Institute.

## 3 Results

### 3.1 Global Trends in Lung Cancer Incidence and Mortality Attributable to Fasting Hyperglycemia

**FIGURE 2:**
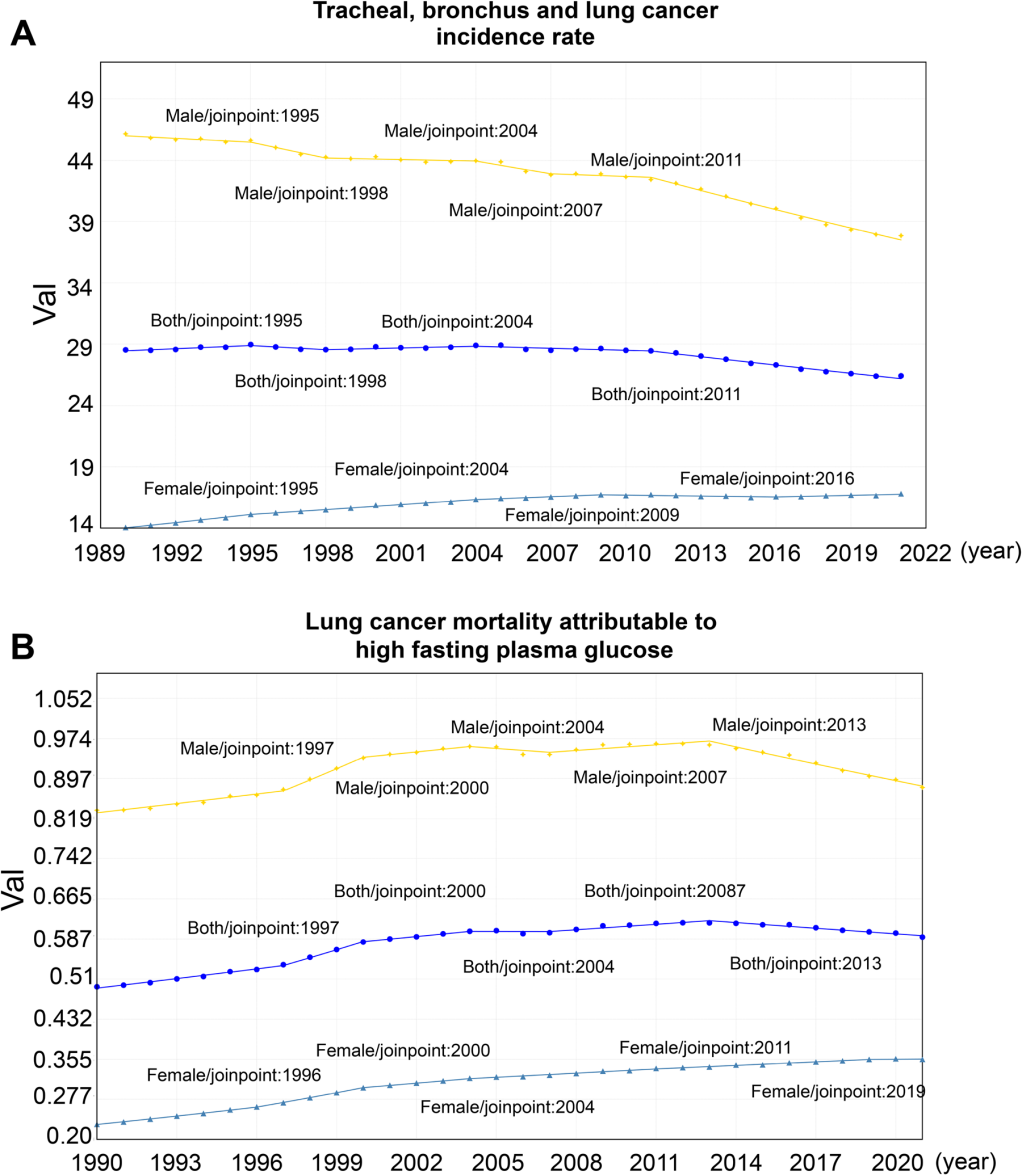
Trends in Lung Cancer Incidence and Mortality Attributable to High Fasting Plasma Glucose. (A) Joinpoint regression modeling of age‐standardized lung cancer incidence rates (1990–2021), based on data from 204 countries/regions and 811 areas (Global Burden of Disease 2021). Annual percent change (APC) and average annual percent change (AAPC) with 95% confidence intervals (CI) are presented, with statistical significance assessed using permutation tests (*p* < 0.05 indicating significance). (B) Joinpoint regression modeling of age‐standardized lung cancer mortality rates attributable to fasting hyperglycemia (1990–2021), based on the same dataset (*n* = 204 countries/regions, 811 areas). APC and AAPC with 95% CI are shown, with significance determined by permutation tests (*p* < 0.05, denoted by *). Data are presented as rates per 100 000 population.

The results of the Joinpoint regression analysis (Figure 2A; Table S1) indicated that, from 1990 to 2021, the overall incidence rate of lung cancer for both men and women showed a decreasing trend, with an age‐standardized incidence rate AAPC of −0.267 (95% CI: −0.363 to −0.17, *p* < 0.001). However, it was evident that the trend differed between genders. For men, the incidence rate decreased (AAPC = −0.657, 95% CI: −0.756 to −0.558, *p* < 0.001), whereas for women, the incidence rate increased over the 30‐year period (AAPC = 0.578, 95% CI: 0.509 to 0.647, *p* < 0.001). Given that diabetes may increase lung cancer incidence and mortality [8, 40, 41], we also conducted a regression analysis on lung cancer mortality attributable to fasting hyperglycemia during the same period (Figure 2B; Table S2). The results showed that the age‐standardized mortality rate AAPC for both genders was 0.607 (95% CI: 0.468 to 0.746, *p* < 0.001), with increasing trends for both men (AAPC: 0.195, 95% CI: 0.036 to 0.355, *p* = 0.016) and women (AAPC: 1.43, 95% CI: 1.339 to 1.52, *p* < 0.001). Notably, the age‐standardized mortality rate for lung cancer attributable to fasting hyperglycemia in men began to decline from 2013 to 2021 (APC: −1.171, 95% CI: −1.287 to −1.054, *p* < 0.001), while no such declining trend was observed for women. In short, while the overall global incidence of lung cancer has shown gender‑specific trends, our regression analysis demonstrated that fasting hyperglycemia is associated specifically with increasing lung cancer mortality, particularly among females.

### 3.2 Exploring the Causal Relationship between CTSH and Lung Cancer Based on Multiple Databases

**FIGURE 3:**
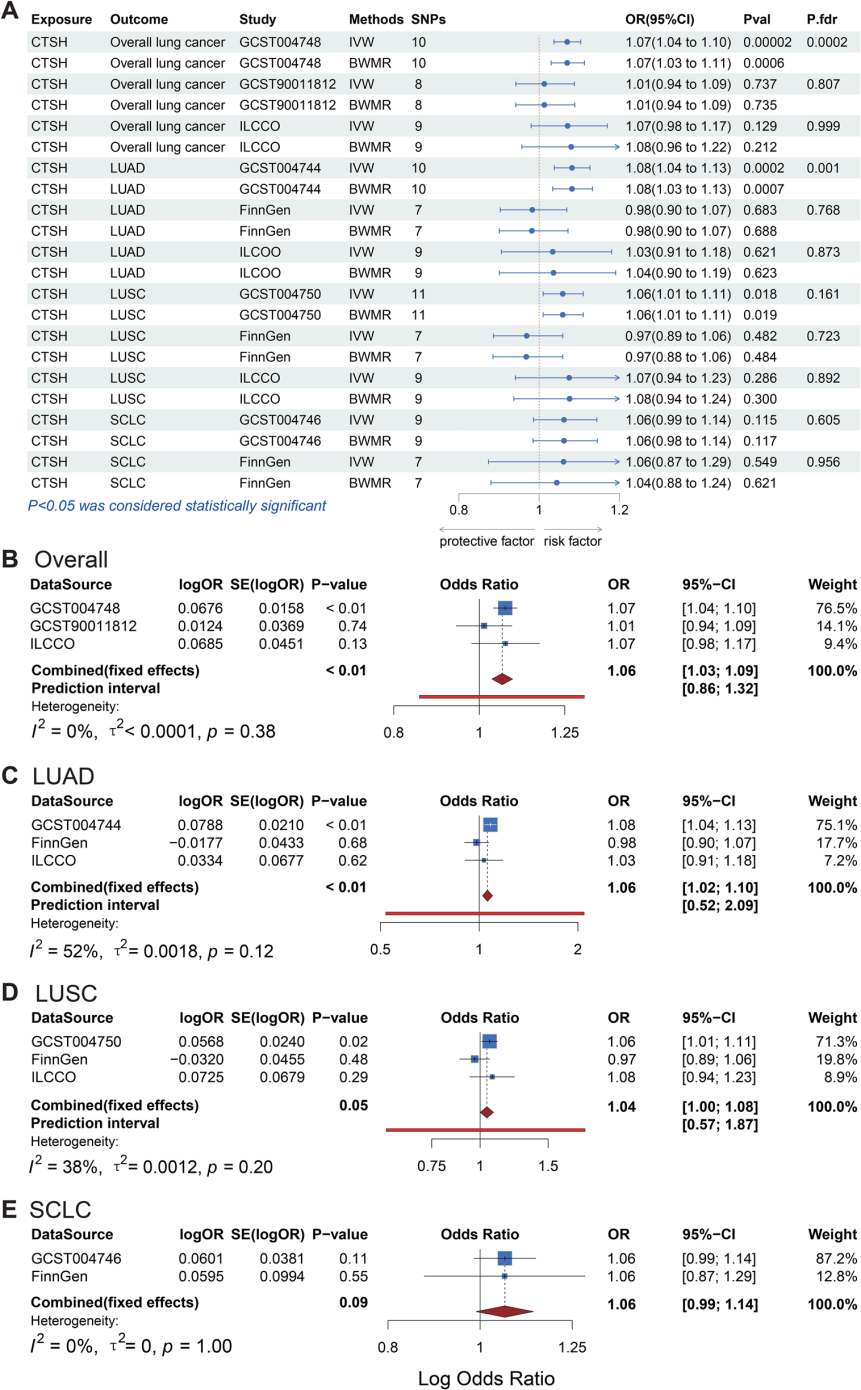
Comprehensive Mendelian Randomization and Meta‐Analysis of Cathepsin H Across Overall Lung Cancer and Its Subtypes. (A) MR results using the inverse variance weighted (IVW) method across datasets (McKay et al.: *n* = 29,863 cases, 55 586 controls; Rshkin et al.: *n* = 2,485 cases, 410 350 controls; FinnGen: *n* = 3642 cases, 287 137 controls); ILCCO: *n* = 11 348 cases, 15 861 controls. Two‐sided IVW tests with false discovery rate (FDR) correction were used, with *p* < 0.05 indicating significance. (B–E) Forest plots showing the results of meta‐analysis of overall lung cancer, LUAD, LUSC, and SCLC in order, using fixed‐ or random‐effects models based on Cochran's Q test for heterogeneity (*p* > 0.05 for fixed‐effects).

Since a high‐glucose environment could promote the increase of tissue proteases through various mechanisms [42, 43]. We further investigated the relationship between CTSH and lung cancer using CTSH protease GWAS data from the INTERNAL study. After removing SNPs with *p* > 1e‐5 and F < 10, we conducted two‐sample MR analyses to explore the potential associations between CTSH and lung cancer and its subtypes (Figure 3A). Initially, we found that high levels of CTSH were associated with a modest increase in the risk of overall lung cancer in the GWAS data by McKay JD et al. (IVW: p = 2 × 10^−^⁵, OR = 1.07, 95% CI = 1.04–1.10). This significance persisted after applying the BWMR algorithm (p = 5.81 × 10^−4^) and FDR correction (p = 1.80 × 10^−4^), with consistent results across all analysis directions. However, MR analyses using GWAS data from Rshkin et al. (IVW: p = 0.737, OR = 1.01, 95% CI = 0.94–1.09) and ILCCO (IVW: p = 0.129, OR = 1.07, 95% CI = 0.98–1.17) cohorts produced inconsistent results, with no significant findings after BWMR and FDR corrections. This highlights the potential for heterogeneity in the association between CTSH and lung cancer risk, likely due to differences in population characteristics and study methodologies. To enhance the credibility of our findings, we performed a meta‐analysis of the IVW results from three datasets, which revealed an OR = 1.06 (95% CI = 1.03–1.09, *P* < 0.01), further confirming that high CTSH levels increased lung cancer risk (Figure 3B).

Next, we focused on the relationship between CTSH and specific lung cancer subtypes. In the GWAS data by McKay JD et al., high CTSH levels were associated with increased risks of lung adenocarcinoma (IVW: p = 1.72 × 10^−4^, OR = 1.08, 95% CI = 1.04–1.13) and lung squamous cell carcinoma (IVW: p = 1.79 × 10^−^^2^, OR = 1.06, 95% CI = 1.01–1.11). Lung adenocarcinoma maintained strong significance after BWMR algorithm validation (p = 7.06 × 10^−4^) and FDR correction (p = 1.55 × 10^−3^). Although lung squamous cell carcinoma showed good results with the BWMR algorithm (p = 0.02), it lost significance after FDR correction. All results were consistent with the IVW analysis direction. We then conducted MR analyses on GWAS data for lung adenocarcinoma and lung squamous cell carcinoma from the FinnGen and ILCCO databases. Surprisingly, we found no significant causal effects between CTSH and lung adenocarcinoma or lung squamous cell carcinoma, with p‐values remaining > 0.05 after BWMR and FDR corrections. Meta‐analysis of the IVW results from three databases showed a strong association between CTSH and lung adenocarcinoma (OR = 1.06, 95% CI = 1.02–1.10, *P* < 0.01), while lung squamous cell carcinoma lost significance (OR = 1.04, 95% CI = 1.00–1.08, P = 0.05) (Figure 3C,D).

Finally, we analyzed the association between CTSH and small‐cell lung cancer. The GWAS data from McKay JD et al. (IVW: p = 0.11, OR = 1.06, 95% CI = 0.99–1.14) and the FinnGen database (IVW: p = 0.55, OR = 1.06, 95% CI = 0.87–1.29) showed no significant results. All analysis methods were consistent with IVW, and BWMR algorithm validation and FDR correction also showed no significance. Meta‐analysis of the two databases confirmed the absence of a causal relationship between CTSH levels and small cell lung cancer (OR = 1.06, 95% CI = 0.99–1.14, P = 0.09) (Figure 3E). To further evaluate the robustness of heterogeneity, we conducted a leave‐one‐dataset‐out sensitivity analysis, which showed consistent effect direction (OR≈1.05–1.07), but excluding certain datasets significantly reduced I^2^, suggesting specific studies as major sources of heterogeneity. For small cell lung cancer, with only two datasets, I^2^ was unavailable after exclusion (Figure S12, Table S10). These results indicate a modest effect size with heterogeneity across datasets, likely due to differences in sample size, population diversity, and GWAS study design. SNP heterogeneity and pleiotropy tests, including Cochran's Q, MR‐Egger intercept, and MR‐PRESSO, showed no heterogeneity or horizontal pleiotropy in the MR analysis. The MR analysis results and leave‐one‐out sensitivity analysis are shown in Figures S1–S4 and Table S3. Additionally, reverse MR analysis of GWAS data for CTSH and lung cancer subtypes revealed no reverse causality (Table S4). In our study, it was determined that CTSH facilitated the progression of both overall lung cancer and lung adenocarcinoma, whereas it exhibited no significant association with other lung cancer subtypes.

### 3.3 Exploring the Mediating Role of 233 Metabolic Traits in Lung Adenocarcinoma

**FIGURE 4:**
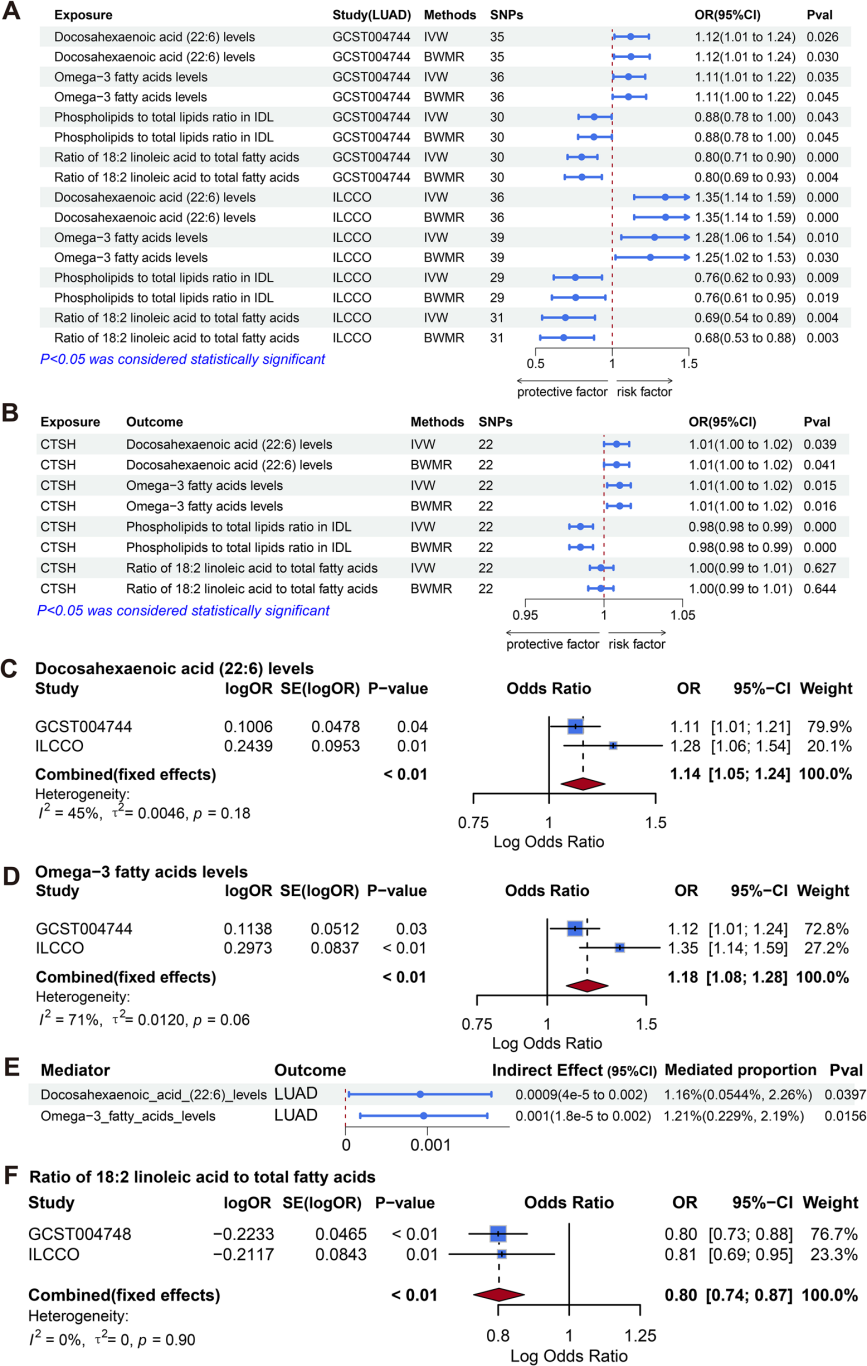
Two‐Step Mendelian Randomization Analysis to Elucidate the Mediating Influence of 233 Metabolic Traits in the Causal Pathway Between CTSH and Lung Adenocarcinoma. (A) MR analysis of 233 metabolic traits associated with lung adenocarcinoma, using GWAS data (McKay et al.: *n* = 11,273 cases, 55 586 controls; ILCCO: *n* = 3,442 cases, 15 861 controls; metabolic traits: *n* = 136 016 participants). Two‐sided IVW tests with FDR correction were used (*p* < 0.05). (B) MR analysis of CTSH and four metabolic traits (*n* = 3,301 for CTSH; *n* = 136 016 for traits), using two‐sided IVW and Bayesian weighted MR (BWMR) tests (*p* < 0.05). (C, D) Meta‐analysis of docosahexaenoic acid (DHA, 22:6) levels (C) and omega‐3 fatty acid levels (D), using fixed‐ or random‐effects models based on Cochran's Q test (*p* < 0.05). (E) Mediation analysis of DHA (22:6) and omega‐3 fatty acid levels, showing indirect effects with 95% CI, assessed via the product of coefficients method and delta method for standard errors (*p* < 0.05). (F) Meta‐analysis of the ratio of 18:2 linoleic acid to total fatty acids for overall lung cancer, using two‐sided IVW tests (*p* < 0.05).

Based on the previous findings, it was evident that there was a significant association between CTSH and lung cancer, particularly with lung adenocarcinoma. We further utilized the recently released GWAS data for 233 metabolites to investigate their mediating roles between CTSH and lung adenocarcinoma. Initially, we screened the metabolite GWAS data from the GWAS catalog using stringent criteria (*p* < 5e^−8^ and F > 10) to identify eligible SNPs. Subsequently, we employed MR analysis to explore the causal relationships between these metabolites and lung adenocarcinoma (Figure 4A). In the GWAS data for lung adenocarcinoma from McKay JD et al. and the ILCCO database, we identified causal relationships with 16 and 17 distinct metabolic traits, respectively. However, due to insufficient significance after FDR correction, we intersected the results from both groups to enhance stability. Finally, we identified four metabolite traits with highly credible causal relationships with lung adenocarcinoma: Docosahexaenoic acid (DHA) (22:6) levels, Omega‐3 fatty acids levels, Phospholipids to total lipids ratio in IDL, and Ratio of 18:2 linoleic acid to total fatty acids (Table S5). All these metabolites had p‐values < 0.05 in BWMR analysis.

We then explored the causal relationships between CTSH and these four metabolite traits. The findings indicated notable causal associations between CTSH and three metabolite characteristics (Figure 4B): DHA (22:6) levels (IVW: p = 0.039, OR = 1.01, 95% CI = 1.00–1.02), Omega‐3 fatty acids levels (IVW: p = 0.015, OR = 1.01, 95% CI = 1.00–1.02), and Phospholipids to total lipids ratio in IDL (IVW: p = 9.13×10^−^⁵, OR = 0.99, 95% CI = 0.98–0.99), with strong significance remaining after BWMR analysis and FDR correction. However, no significant relationship was found between CTSH and the Ratio of 18:2 linoleic acid to total fatty acids. All these analyses passed heterogeneity and pleiotropy tests (Table S6), with MR analysis results shown in Figures S5–S7. Notably, reverse MR analysis showed a reverse causal link between the Phospholipids to total lipids ratio in IDL and lung adenocarcinoma (Tables S7 and S8). Finally, we identified two metabolite traits (DHA (22:6) levels and Omega‐3 fatty acids levels) as mediators for further investigation. To further validate the results of the previous MR analysis. Meta‐analysis of the IVW results from the two data sources confirmed significant associations between these two metabolites and lung adenocarcinoma (Figure 4C,D). The results showed DHA (22:6) levels: OR = 1.18 (95% CI = 1.08–1.28, *P* < 0.01), and Omega‐3 fatty acids levels: OR = 1.14 (95% CI = 1.05–1.24, *P* < 0.01).

Subsequently, we utilized a two‐step method to analyze the mediating roles of these two metabolite traits in the causal pathway from CTSH to lung adenocarcinoma, using the delta method to estimate standard errors. We found that the indirect effect of DHA (22:6) levels in the causal pathway from CTSH to lung adenocarcinoma was 0.0009 (95% CI 4.3e‐5‐0.002, p = 0.0397), with a corresponding mediation proportion of 1.16% (95% CI 0.05% ‐ 2.26%). Similarly, the indirect effect of Omega‐3 fatty acids levels was 0.001 (95% CI 1.8e‐4‐0.002, p = 0.0156), with a mediation proportion of 1.21% (95% CI 0.23–2.19%) (Figure 4E). In a word, utilizing various validation techniques, we determined that levels of DHA (22:6) and Omega‐3 fatty acids played a mediating role in the progression of lung adenocarcinoma. However, in previous studies reported, DHA (22:6) and Omega‐3 fatty acids played anti‐inflammatory and anti‐tumor roles [44]. Therefore, our study was likely to provide a new perspective on the treatment of lung cancer.

### 3.4 Exploring the Mediating Role of 233 Metabolic Traits in Overall Lung Cancer

Subsequently, given the potential variations in the pathogenesis of distinct lung cancer subtypes, we conducted an in‐depth investigation to elucidate the specific metabolic characteristics that mediate the relationship between CTSH and overall lung cancer. Screening criteria consistent with lung adenocarcinoma. In the overall lung cancer GWAS data from McKay JD et al. and the ILCCO database, we identified causal associations with five and two different metabolic traits, respectively. Due to the lack of significance of the FDR correction, we similarly took the intersection of the results of the two groups to improve stability. A potential causal relationship between the Ratio of 18:2 linoleic acid to total fatty acids and overall lung cancer was finally established with reverse validation and BWMR (*p* < 0.05). In addition, to make the results more rigorous, we likewise performed a Meta‐analysis of the results, which suggested a negative correlation association between the Ratio of 18:2 linoleic acid to total fatty acids and overall lung cancer (Figure 4F). However, we subsequently utilized MR and did not find a causal relationship between CTSH and the Ratio of 18:2 linoleic acid to total fatty acids. All of these analyses passed heterogeneity and multiple validity tests (Tables S5–S7). In summary, our analysis did not identify a mediating role for the 233 metabolite traits in the context of overall lung cancer. However, the Ratio of 18:2 linoleic acid to total fatty acids demonstrated a potential association with a reduced risk of developing overall lung cancer. To further validate CTSH expression differences in key macrophage subtypes, we extracted mo‐Mac (*n* = 5452) and Alveolar Mac (*n* = 11 824) from the GSE131907 dataset to create a new Seurat object and generated an integrated figure (Figure S13). The UMAP shows cell distribution, while the violin plot displays CTSH expression levels and their comparison. The mo‐Mac vs Alveolar Mac contrast yields a logFC of 0.319, FDR of 5.059504e‐09 (p = 1.707331e‐13, two‐sided Wilcoxon rank‐sum test, *p* < 0.0001, ^****^ indicates significance), showing significantly higher CTSH expression in mo‐Mac than in Alveolar Mac.

### 3.5 Co‐Localization Analysis: Additional Validation of Positive MR Results

The co‐localization analysis revealed that overall lung cancer, lung adenocarcinoma, and CTSH shared a common causal variant at chromosome 15 rs34593439 with a PPH4 value of 0.99 (PPH4 > 0.8), indicating high support for shared causal variants. Additionally, CTSH and Omega‐3 fatty acids levels at chromosome 11 rs174564 had a PPH4 of 0.58, and DHA (22:6) levels and lung adenocarcinoma at chromosome 11 rs2524296 had a PPH4 of 0.76, providing moderate evidence for co‐localization (PPH4 > 0.5). Overall, lung cancer and the Ratio of 18:2 linoleic acid to total fatty acids shared a common causal variant on chromosome 11, rs97384, with a PPH4 value of 1 (PPH4 > 0.8). No satisfactory H4 hypothesis was found for the remaining co‐localization results (Table S9). Significant chromatin variation may be present in tumor tissue compared to normal tissue [45]. It was noteworthy that colocalization analysis, as a necessary but not sufficient condition for MR analysis, did not invalidate MR results even if negative [46]. Collectively, more vigorously supporting the significant roles of DHA (22:6) levels and Omega‐3 fatty acids levels in CTSH‐related lung adenocarcinoma.

### 3.6 Investigating the Expression and Function of CTSH at the Cellular and Genetic Levels in Lung Cancer Tissues

**FIGURE 5:**
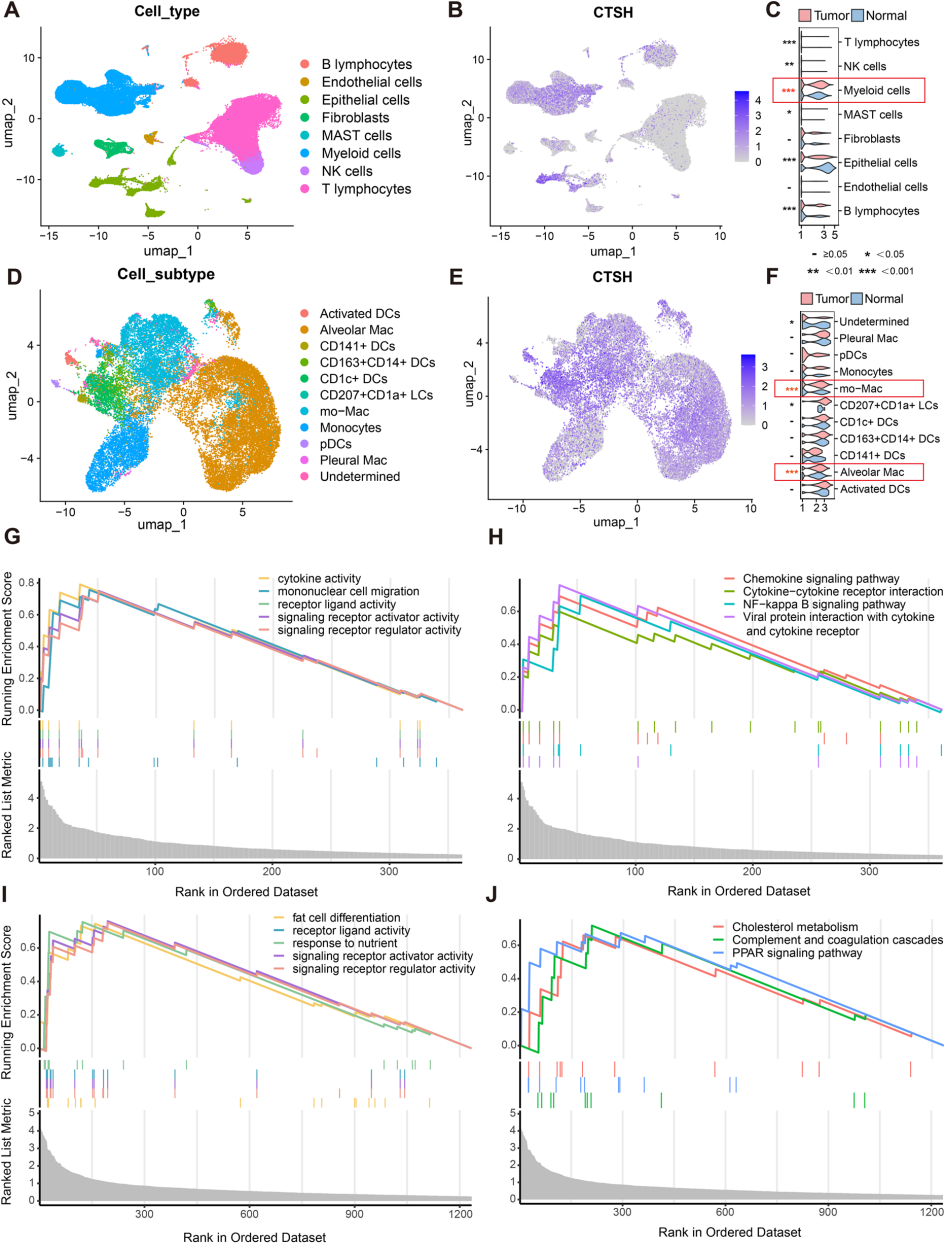
Single‐Cell Transcriptome and Functional Enrichment Analyses of CTSH in Lung Adenocarcinoma. (A) Umap plots showing single‐cell annotation results of LUAD and normal lung tissue samples (*n* = 89 902 cells after quality control). (B, C) Umap plot (B) and violin plot (C) showing CTSH‐enriched cell clusters. (D) Further dimensionality reduction and cell annotation of myeloid cells (*n* = 24,093 cells). (E, F) Umap plot (E) and violin plot (F) exhibiting the enrichment of CTSH in specific cell types. Two‐sided Wilcoxon rank‐sum tests assessed expression differences (The figure shows how statistical differences are represented). (G, H) GSEA analysis of GO and KEGG for mo‐Mac. (I, J) GSEA analysis of GO and KEGG for Alveolar Mac.

Initial dimensionality reduction and annotation of scRNA data identified eight cell types (Figure 5A) and highlighted differences between Tumor and Normal groups (Figure S8A,B). Cells with an expression level of genes ranging from 500 to 4000 and mitochondrial gene content percentage below 10% were retained. The final number of retained cells was 89 902. The target gene CTSH was primarily concentrated in Myeloid cells and Epithelial cells (Figure 5B,C). Apart from macrophages, most myeloid cells (e.g., neutrophils, mast cells, etc) can express CTSH [47, 48]. We extracted the Myeloid cells cluster for further dimensionality reduction and annotation, identifying 11 cell types with 24 093 cells (Figure 5D). CTSH expression was observed across various Myeloid cell subgroups (Figure 5E), with noticeable expression differences in “mo‐Mac”, “CD207+CD1a+ LCs”, “Alveolar Mac”, and no significant difference (*p*> 0.05) observed in pDCs (Figure 5F). These subgroups contained 120, 5452, 90, and 11 824 cells, respectively. Thus, “mo‐Mac” and “Alveolar Mac” were selected for further study, with “mo‐Mac” enriched in the Tumor group and “Alveolar Mac” in the Normal group (Figure S8C,D). Subsequent analyses excluded the “Undetermined” cell type.

Using the FindAllMarkers function, we compared Myeloid cell subgroups to identify highly variable genes, selecting those from “mo‐Mac” and “Alveolar Mac” for functional enrichment analysis. Detailed enrichment results of GO and KEGG were presented in Supporting Information (Figure S11). By virtue of the GSEA, GO enrichment results indicated that highly variable genes in “mo‐Mac” (Figure 5G) were enriched in cellular interactions and signaling processes, highly likely to influence the functions of macrophages and their involvement in immune and inflammatory processes.Similarly, KEGG enrichment results included “Chemokine signaling pathway”, “Cytokine−cytokine receptor interaction”, “NF−kappa B signaling pathway” and “Viral protein interaction with cytokine and cytokine receptor” (Figure 5H). For GO enrichment results of “mo‐Mac” and “Alveolar Mac”, they had the same enriched pathways, “signaling receptor activator activity”, “signaling receptor regulator activity” (Figure 5I). KEGG enrichment results of “Alveolar Mac” consisted of “Cholesterol metabolism”, “Complement and coagulation cascades” and “PPAR signaling pathway”, focusing on organismal functions and metabolism (Figure 5J). Compared to the enriched KEGG pathways in mo‐Mac, these pathways appeared to be more closely related to fundamental physiological functions in the body rather than intense immune‐inflammatory responses.

**FIGURE 6:**
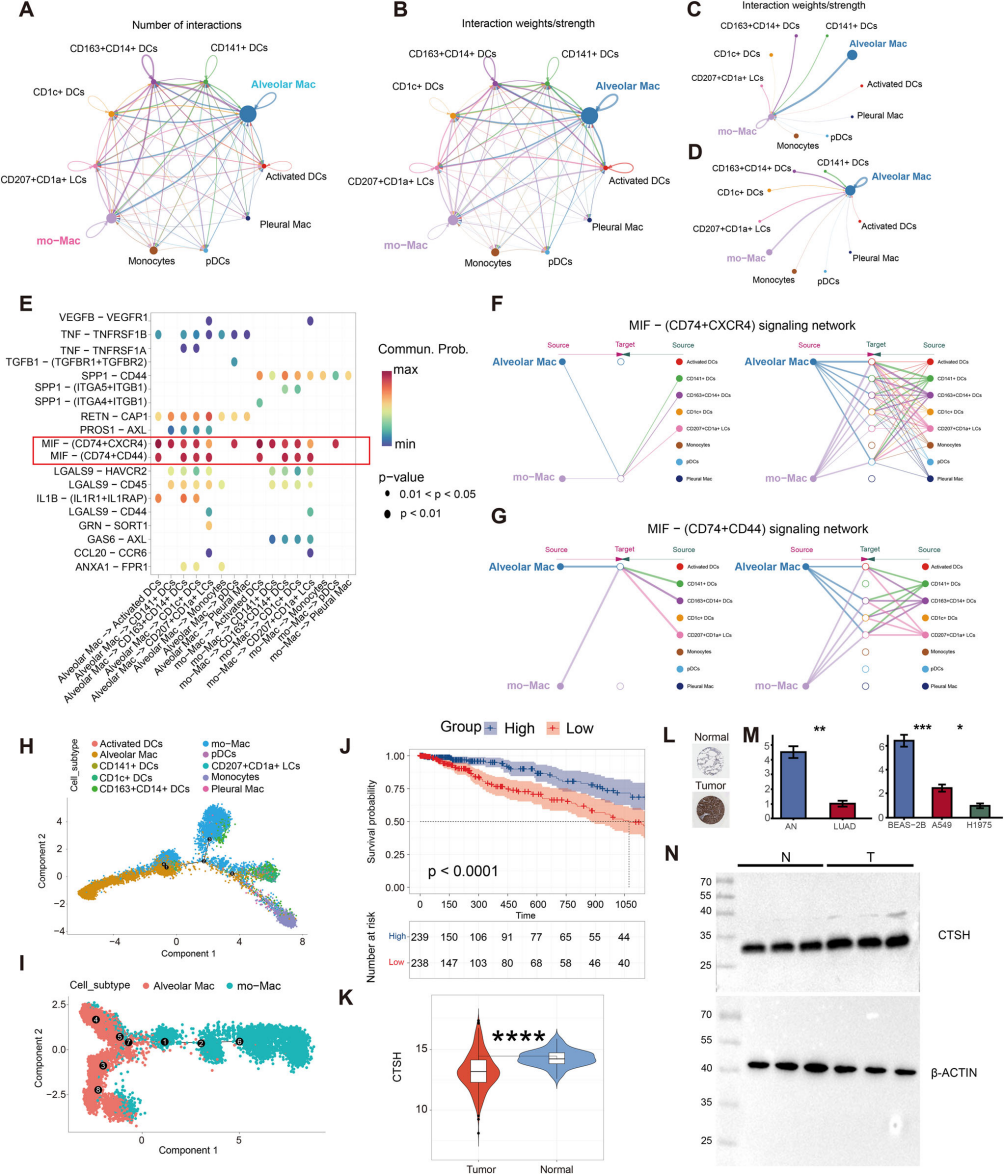
Integrative Analyses of Cellular Interactions, Signaling Networks, and Prognostic Significance of CTSH in Lung Adenocarcinoma. (A, B) Number (A) and weights (B) of interaction among major cell types (*n* = 89 902 cells). (C, D) Interaction weights involving mo‐Mac (*n* = 5,452 cells) (C) and Alveolar Mac (*n* = 11,824 cells) (D) as signal recipients. (E) The correlation between specific cell communications and signaling pathways. (F, G) The cell communication performance of target cell types in MIF‐(CD74+CXCR4) signaling network (F) and MIF‐(CD74+CD44) signaling network (G). (H, I) Pseudo‐time trajectories of major cell types (H) and myeloid cell subtypes (I). (J) Prognostic evaluation of CTSH in TCGA‐LUAD, using a two‐sided log‐rank test (*p* < 0.0001). (K) Differential expression of CTSH in tumor and normal tissues from TCGA‐LUAD (*n* = 477), assessed by two‐sided Wilcoxon rank‐sum test (*p* < 0.0001, ****). (L) Protein expression of CTSH in tumor and normal tissue from HPA. The blue region represents the nucleus, while the brown region indicates positive gene expression. (M) RT‐qPCR to explore the mRNA levels of CTSH in adjacent normal tissues, lung adenocarcinoma tissues, lung adenocarcinoma cell line A549 and H1975, and normal lung bronchial epithelial cell line BEAS‐2B, assessed by two‐sided t‐test (*p* < 0.05, *; *p* < 0.01, **). (N) Western blotting results of the CTSH expression in lung adenocarcinoma tissues and adjacent normal tissues.

The communication network between cells was performed by calculating the probability and intensity of cell‐cell interactions (Figure 6A,B). Given its complexity, we detected signals emitted by each cell subgroup (Figure S9), focusing on the interaction intensity between “mo‐Mac” and “Alveolar Mac” (Figure 6C,D). Additionally, we displayed the distribution of related cell communications across multiple signaling pathways with these two cell types as signal emitters (Figure 6E). We also calculated the contribution of various ligand‐receptor pairs to cell‐cell communication, finding that the MIF (CD74+CXCR4) and MIF (CD74+CD44) signaling pathways played crucial roles (Figure S10). Overexpression of the chemokine receptor CXCR4 has been found to promote tumor growth, metastasis, and invasion through a variety of mechanisms, including regulation of tumor angiogenesis, immune escape, and the CXCL12/CXCR4 signaling pathway [49, 50]. Therefore, we selected the MIF signaling pathway, designating “mo‐Mac” and “Alveolar Mac” cells as receptors, highlighting their important positions in signal communication (Figure 6F,G). In the MIF (CD74+CXCR4) pathway, “mo‐Mac” acted as the receptor, receiving signals from Alveolar Mac, CD141+DCs, CD163+CD141+DCs, and CD207+CD1a+LCs cells. In the MIF (CD74+CD44) pathway, “Alveolar Mac” served as the receptor, receiving signals from the other cells. To further determine the developmental stages of myeloid cell subgroups, we conducted pseudotime analysis using Monocle2. Both in the overall myeloid cell data (Figure 6H) and in the selected two cell types (Figure 6I), we observed a clear trend of “Alveolar Mac” evolving toward “mo‐Mac.” This was consistent with the general direction of tumor development.

In the TCGA data, the samples with a low expression of CTSH exhibited worse survival in the tumor group (Figure 6J), and CTSH displayed elevated expression in the normal group (Figure 6K). However, analysis of CTSH protein expression levels in the HPA database demonstrated markedly higher expression in the tumor group relative to the normal group (Figure 6L). These results indicate the inconsistent CTSH expression between the transcription level and the protein level. To validate this discrepancy, the RT‐qPCR showed that the relative mRNA expression levels of CTSH were significantly lower in the lung adenocarcinoma specimens and cell lines (A549 and H1975) compared to the normal ones (Figure 6M), whereas the Western blotting revealed the higher expression of CTSH in tumor specimens at the protein level (Figure 6N).

## 4 Discussion

Lung cancer remained one of the most critical threats to human health and life expectancy [51]. From an epidemiological perspective, our study utilized the GBD2021 database to conduct a regression analysis on the incidence of lung cancer patients worldwide from 1990 to 2021. With advancements in immunotherapy for lung cancer [52, 53], we observed a general declining trend in global lung cancer incidence. Notably, since 1990, the age‐standardized incidence rate of lung cancer in females has increased by an average of 0.578% annually. Additionally, our research showed that lung cancer mortality rates due to fasting hyperglycemia have risen in both males and females from 1990 to 2021. Hyperglycemia‐induced oxidative stress and inflammation not only directly damaged cells but also upregulated cathepsins through gene regulation. Thus, it was essential to investigate the potential connection between CTSH and lung cancer.

Fasting hyperglycemia may contribute to elevated CTSH expression via hyperglycemia‐induced oxidative stress and inflammatory signaling pathways. Lysosomal protease activity regulation by glucose has been reported in metabolic studies, providing a mechanistic link between hyperglycemia and CTSH regulation. Therefore, our findings highlight a potential pathway in which metabolic stress may upregulate CTSH, thereby contributing to lung cancer progression. CTSH inhibitors had emerged as potential therapeutic agents for various diseases [54]. Research has shown that certain cysteine cathepsins mediated tumor progression, angiogenesis, and metastasis [55, 56], but studies specifically focusing on CTSH in cancer have remained limited. Although previous MR studies suggested a potential relationship between CTSH and lung cancer, their reliance on a single data source resulted in significant sample bias. Our research, which integrated MR analysis from multiple sources, found a modest association between CTSH and lung cancer risk. However, the effect sizes were small, and results were not consistently replicated across datasets, highlighting the need for further validation and caution in interpreting these findings. To mitigate sample‐related bias, our study integrated MR results from multiple databases for a meta‐analysis and employed colocalization analysis for validation. This comprehensive approach significantly addressed the limitations of previous studies. Ultimately, our study suggests that elevated CTSH levels may be associated with an increased risk of overall lung cancer and lung adenocarcinoma, consistent with the significantly elevated serum CTSH levels observed in histological lung tumor patients [57]. However, the magnitude of this association was modest (meta‑analysis OR ≈ 1.06), and results were not entirely consistent across datasets, with positive findings observed in the McKay cohort but not replicated in FinnGen or ILCCO. These discrepancies suggest that while CTSH may be linked to lung cancer, the effect is small, and further studies with larger sample sizes are needed to confirm these associations. Moreover, no risk association existed between CTSH and squamous cell lung carcinoma or small cell lung cancer. Taken together, while current genetic and expression data suggest a possible pathogenic role of CTSH in lung adenocarcinoma, the evidence remains preliminary, and no definitive consensus has yet been reached. Further mechanistic and experimental validation will be required.

Despite the modest pooled association (OR ≈ 1.06), heterogeneity remains likely, as datasets such as ILCCO and Rashkin showed non‐significant results. These discrepancies may stem from differences in case definitions, population structures, and instrument availability. While we did not perform formal sensitivity analysis omitting entire cohorts, the robustness checks (MR‐PRESSO, Egger intercept, Cochran's Q, leave‐one‐SNP, and leave‐one‐dataset analyses) did not reveal major violations. Therefore, the association should be regarded as modest and hypothesis‐generating, with further replication in larger studies necessary to confirm its validity. Currently, CTSH has already been found to be involved in Talin processing for focal adhesion of migrating PC‐3 cells, suggesting that intracellular protein hydrolysis played a role in regulating cancer cell migration [58]. On the other hand, the T3‐mediated upregulation of extracellular CTSH might result in the activation of matrix metalloproteinases or extracellular signal‐regulated kinases, thereby enhancing cellular migration [59]. However, the precise mechanism by which CTSH influences lung adenocarcinoma is presently unclear and warrants further investigation.

Subsequently, we further analyzed the mediating role of 233 metabolic traits between CTSH and lung adenocarcinoma and overall lung cancer. Following reverse MR screening and Sobel testing, we discovered that omega‐3 fatty acid levels and DHA (22:6) levels mediated the causal relationship between CTSH and lung adenocarcinoma, aligning with previous research findings [60]. However, the mediation proportions were modest, accounting for only ∼1%–2% of the total CTSH effect. Such small effect sizes indicate limited biological contribution, and therefore, these findings should be interpreted with caution, serving as hypothesis‑generating rather than conclusive evidence of a major metabolic pathway. Moreover, although instrumental strength was adequate (all F‑statistics >10), it should be noted that Sobel tests are conservative and have limited precision for detecting small indirect effects, which may partly explain the modest mediation values observed. Future larger GWAS datasets will be required to improve statistical power and validate these pathways.

Further colocalization analysis provided moderate evidence supporting the association of CTSH with omega‐3 fatty acid levels, as well as DHA levels and lung adenocarcinoma. It was noteworthy that previous studies had generally considered omega‐3 fatty acids (particularly DHA) to possess anti‐inflammatory properties [44], with omega‐6 fatty acids primarily promoting inflammation and tumor growth. However, some investigations had suggested that dietary omega‐3 polyunsaturated fatty acids might promote liver metastasis of colon carcinoma in rats, and high plasma DHA levels were associated with an increased risk of prostate cancer [61, 62]. These discrepancies may reflect dietary sources, dosage effects, enzymatic conversion into context‑dependent lipid mediators, or modulation by the tumor microenvironment. The precise mechanisms by which DHA (22:6) and omega‑3 increase lung cancer risk remain to be clarified, and our findings should therefore be regarded as preliminary and hypothesis‑generating [63, 64]. Then, considering the differences in the pathogenesis of various subtypes of lung cancer, we further analyzed overall lung cancer. Unfortunately, no mediating relationship was found. However, as previously reported in the literature that linoleic acid enhances the metabolic adaptive strength and antitumor immunity of CD8+ T cells [65], we also found that the Ratio of 18:2 linoleic acid to total fatty acids was associated with a reduction in overall lung cancer risk and provided a high level of evidence in the co‐localization analysis.

To clarify CTSH's role in lung cancer, we performed single‐cell transcriptome analysis to explore CTSH gene expression in different cell types within lung cancer tissues and conducted pathway enrichment analysis to validate the MR results. It revealed that CTSH was predominantly expressed in myeloid cells and epithelial cells. Specifically, CTSH was enriched in “mo‐Mac” in the tumor group, while it was enriched in “Alveolar Mac” in the normal group, suggesting potential functional differences of CTSH under varying disease states. Functional enrichment analysis of “mo‐Mac” indicated involvement in immune system response, cell activation, intracellular material processing, and immune signaling pathways, whereas “Alveolar Mac” was enriched in granulocyte immune response, intercellular substance transport structures, and oxidoreductase activity processes. Cell‐cell communication analysis revealed the significant roles of “mo‐Mac” and “Alveolar Mac” in multiple signaling pathways, particularly the MIF signaling pathway (CD74+CXCR4 and CD74+CD44). As a chemokine receptor, CXCR4 promoted cancer cell growth, metastasis, and invasion in various cancers [66, 67]. Additionally, pseudotime analysis using Monocle2 revealed an evolutionary trajectory from “Alveolar Mac” to “mo‐Mac”. Finally, transcriptome‐level validation using TCGA data showed higher RNA expression levels of CTSH in the normal group, correlating with better survival rates.

However, protein level analysis from the HPA revealed significantly higher CTSH protein expression in the tumor group, consistent with plasma protein‐level CTSH expression in the MR analysis. This kind of inconsistent expression levels between RNA transcription and protein translation can often be observed across varieties of cancers [68, 69]. Since mRNA levels do not necessarily correlate with protein abundance due to post‐transcriptional regulation, protein translation efficiency, and protein degradation dynamics. Furthermore, cohort differences and methodological variations, such as RNA‐seq quantification versus immunohistochemistry scoring, may also contribute to these inconsistencies. Importantly, the protein‐level upregulation observed in tumors was in line with our MR‐based genetic evidence, suggesting that proteomic alterations may better capture CTSH's functional role. We also performed validation using PCR and WB methods, which revealed opposing expression trends of CTSH at the RNA and protein levels. For example, TEM8 protein expression was significantly higher in triple‐negative breast cancer tissues than in paraneoplastic tissues and other subtypes, and further studies have confirmed that TEM8 indeed promotes tumor progression. However, at the transcriptome level, the mRNA expression level of the TEM8 gene was not significantly elevated and was even lower than that in breast cancer tissues of the Luminal subtype [69]. Protein level changes were not solely dependent on mRNA levels, but are also related to post‐transcriptional modifications, protein translation, and degradation, etc. Studies have shown that cancer cells could increase specific protein levels by enhancing mRNA translation efficiency or reducing protein degradation [70, 71]. For example, in Chinese patients with non‐small cell lung cancer, combining genomic and transcriptomic analysis showed that the subgroup with homologous recombination deficiency has a worse prognosis and unique molecular traits [72]. This underscores the vital role of multi‐omics approaches in unraveling the complex regulatory mechanisms of lung cancer. Hence, these complex regulatory mechanisms could precisely serve as targets for future therapeutic interventions.

This study innovatively integrated multiple analytical methods, including regression model analysis, MR, and scRNA‐seq, to provide multi‐faceted evidence for understanding the relationship between CTSH and lung cancer. Additionally, the study leveraged publicly available data from multiple databases, enhancing the reliability and representativeness of the results. Through multi‐level validation methods such as meta‐analysis and Bayesian colocalization analysis, the robustness of the study's conclusions was reinforced. Single‐cell transcriptome analysis also offered insights into the expression and function of CTSH in different cell types, elucidating its specific role within the tumor microenvironment. However, the MR study had some limitations. First, despite utilizing several databases, data bias might still exist, and the scope of cases and populations covered by the data needs to be broadened. Second, due to limitations in the available data, gender‐stratified analysis could not be performed, potentially overlooking differences between sexes. Lastly, although the study's metabolic traits and lung adenocarcinoma were subjected to multiple validations, the p‐values were not significant after FDR correction. In addition, our study had not yet explored the function of CTSH in lung cancer at the macrophage level, indicating that further experimental studies were required to confirm these findings. At present, the biological mechanisms between CTSH and lipid metabolism are still poorly studied. Future research should urgently delve into the mechanisms by which CTSH and lipid metabolic traits collectively influence lung cancer development. Furthermore, a comprehensive analysis of the biological mechanisms underlying lung cancer should be conducted through the integration of multi‐omics approaches.

## 5 Conclusions

Our study pioneered a regression analysis of global lung cancer data from the GBD database (1990–2021) and stood as the first to comprehensively examine CTSH and lung cancer through multi‐dimensional, multi‐database, and multi‐validation approaches. This study suggests a possible link between CTSH and lung adenocarcinoma, with DHA (22:6) and Omega‐3 fatty acids identified as potential mediators. Given the modest effect sizes and inconsistencies across datasets, these findings should be regarded as preliminary and interpreted with caution. Even so, they provide important insights into lung cancer biology and open new perspectives for biomarker discovery and targeted therapy, with the potential to improve patient prognosis in clinical practice.

## Author Contributions

Chenghu Song: writing ‐ original draft, software, visualization. Weici Liu: writing ‐ original draft and formal analysis. Zhao He: writing ‐ original draft, software, formal analysis. Jiwei Liu: investigation and data curation. Ruixin Wang: investigation and data curation. Lei Wu: investigation and software. Yize Wang: investigation and software. Mingfeng Zheng: conceptualization, supervision, and funding acquisition. Dong Tian: conceptualization, methodology, and supervision. Wenjun Mao: conceptualization, methodology, resources, funding acquisition, and writing ‐ review & editing. Wenjun Mao is designated as the “corresponding author” and is responsible for the integrity of the work from inception to publication.

## Funding

This work was partially funded by the Natural Science Foundation of Jiangsu Province (BK20210068), the Mega‐project of Wuxi Commission of Health (Z202216), and the Top Talent Support Program for Young and Middle‐aged People of Wuxi Health Commission (BJ2023014).

## Conflicts of Interest

The authors declare no conflicts of interest.

## Peer Review

The peer review history for this article is available in the Supporting Information for this article.

## Supporting information




**Supporting File**: ggn270014‐sup‐0001‐SuppMat.docx

Supporting file: ggn270014‐sup‐0002‐FigureS1.pdf

Supporting file: ggn270014‐sup‐0003‐FigureS2.pdf

Supporting file: ggn270014‐sup‐0004‐FigureS3.pdf

Supporting file: ggn270014‐sup‐0005‐FigureS4.pdf

Supporting file: ggn270014‐sup‐0006‐FigureS5.pdf

Supporting file: ggn270014‐sup‐0007‐FigureS6.pdf

Supporting file: ggn270014‐sup‐0008‐FigureS7.pdf

Supporting file: ggn270014‐sup‐0009‐FigureS8.pdf

Supporting file: ggn270014‐sup‐0010‐FigureS9.pdf

Supporting file: ggn270014‐sup‐0011‐FigureS10.pdf

Supporting file: ggn270014‐sup‐0012‐FigureS11.pdf

Supporting file: ggn270014‐sup‐0013‐FigureS12.pdf

Supporting file: ggn270014‐sup‐0014‐FigureS13.pdf

Supporting file: ggn270014‐sup‐0015‐Table1.xls

Supporting file: ggn270014‐sup‐0016‐Table2.xls

Supporting file: ggn270014‐sup‐0017‐Table3.xls

Supporting file: ggn270014‐sup‐0018‐Table4.xls

Supporting file: ggn270014‐sup‐0019‐Table5.xls

Supporting file: ggn270014‐sup‐0020‐Table6.xls

Supporting file: ggn270014‐sup‐0021‐Table7.xls

Supporting file: ggn270014‐sup‐0022‐Table8.xls

Supporting file: ggn270014‐sup‐0023‐Table9.xls

Supporting file: ggn270014‐sup‐0024‐Table10.xls

Supporting file: ggn270014‐sup‐0025‐Table11.xls

**Supplementary Information**: Record of Transparent Peer Review

## Data Availability

The datasets presented in this study can be found in the online repository. Data URLs: cathepsin H from IEU OpenGWAS project (mrcieu.ac.uk); 233 metabolic profiles can be downloaded from the GWAS Catalog (study accession numbers: GCST90301941 ‐ GCST90302173: GWAS Catalog (ebi.ac.uk)); GWAS statistics for lung cancer and its subtypes were obtained from the FinnGen database (FinnGen: an expedition into genomics and medicine | FinnGen) in addition to the IEU, GWAS Catalog. All codes used in the study are available on request from the corresponding authors.
