# Supplementary material for: Identification of Cathepsin H and Metabolic Traits as Potential Biomarkers for Lung Cancer by Mendelian Randomization and Single‐Cell Transcriptomics
Source: Adv Genet (Hoboken). 2025 Nov 14;6(4):e00012. doi: 10.1002/ggn2.202500012 (PMC12747557; doi:10.1002/ggn2.202500012)
Supplement: Supplementary file 1 — Supporting File: ggn270014‐sup‐0001‐SuppMat.docx [file GGN2-6-e00012-s002.docx]

**Supplementary figure and table legends**

**Supplementary Figure 1.** MR analysis of overall lung cancer: scatterplots, forest plots, leave-one-out plots.

**Supplementary Figure 2.** MR analysis of LUAD: scatterplots, forest plots, leave-one-out plots.

**Supplementary Figure 3.** MR analysis of LUSC: scatterplots, forest plots, leave-one-out plots.

**Supplementary Figure 4.** MR analysis of SCLC: scatterplots, forest plots, leave-one-out plots.

**Supplementary Figure 5.** MR analysis of metabolic traits and lung adenocarcinoma (GCST004744): scatterplot, forest plot, leave-one-out plot.

**Supplementary Figure 6.** MR analysis of metabolic traits and lung adenocarcinoma (ILCCO): scatterplot, forest plot, leave-one-out plot.

**Supplementary Figure 7.** MR analysis of CTSH and metabolic traits: scatterplots, forest plots, leave-one-out plots.

**Supplementary Figure 8**. (**A-B**) Variations in major cell types (**A**) and CTSH enrichment (**B**) between tumor and normal tissues. (**C-D**) Discrepancies in myeloid cell subtypes (**C**) and CTSH enrichment (**D**) between tumor and normal tissue.

**Supplementary Figure 9.** Interaction weights of other myeloid cell subtypes as signal recipients.

**Supplementary Figure 10.** The contributions of various signaling pathways to cell communication.

**Supplementary Figure 11. (A-B)** GO functional enrichment analysis for mo-Mac and Alveolar Mac. **(C)** KEGG functional enrichment analysis of high variant genes in mo-Mac and Alveolar Mac.

S**upplementary Figure 12.** Leave-One-Dataset-Out Sensitivity Analysis for Meta-Analysis of CTSH and Lung Cancer Outcomes.

Data are presented as odds ratios (OR) with 95% confidence intervals (CI) in forest plots for overall lung cancer, LUAD, LUSC, and SCLC. Each panel shows the original meta-analysis results and results from excluding one dataset at a time. For SCLC, with only two datasets, I² was unavailable after exclusion.

**Supplementary Figure 13.** Integrated UMAP and Violin Plot for CTSH Expression in Key Macrophage Subtypes. UMAP plot showing cell distribution of mo-Mac (n = 5,452) and Alveolar Mac (n = 11,824) subtypes. Violin plot exhibiting CTSH enrichment in mo-Mac versus Alveolar Mac. The mo-Mac vs Alveolar Mac contrast yields logFC = 0.319, FDR = 5.059504e-09 (p = 1.707331e-13), assessed by two-sided Wilcoxon rank-sum test, with p < 0.0001 considered significant (****). Significance denoted by **** (p < 0.0001).

**Supplementary Table 1**. Trends in global tracheal, bronchus and lung cancer incidence over the period 1990-2021 (GBD2021).

**Supplementary Table 2**. Global trends in lung cancer mortality attributable to fasting hyperglycemic factors over the period 1990-2021 (GBD2021).

**Supplementary Table 3**. Mendelian randomization analysis of the causal effect of CTSH on lung cancer and among its subtypes and its sensitivity analysis.

**Supplementary Table 4**. Reverse Mendelian randomization: effect of lung cancer subtype on CTSH.

**Supplementary Table 5**. Mendelian and sensitivity analyses: metabolic traits potentially associated with lung adenocarcinoma as well as overall lung cancer.

**Supplementary Table 6**. Mendelian and sensitivity analyses: causal effects of CTSH on potential metabolic traits.

**Supplementary Table 7**. Reverse Mendelian randomization: effect of lung adenocarcinoma as well as overall lung cancer on potential metabolic traits.

**Supplementary Table 8**. Reverse Mendelian randomization: effects of four metabolic traits on CTSH.

**Supplementary Table 9.** Bayesian co-localization analysis.

**Supplementary Table 10.** Quantitative results of Leave-One-Dataset-Out sensitivity analysis for Meta-Analysis of CTSH and lung Cancer outcomes.

**Supplementary Table 11.** Compact SNP Table for Mendelian Randomization Analysis of CTSH and Metabolic Traits.
