# Supplementary material for: Identification of Cathepsin H and Metabolic Traits as Potential Biomarkers for Lung Cancer by Mendelian Randomization and Single‐Cell Transcriptomics
Source: Adv Genet (Hoboken). 2025 Nov 14;6(4):e00012. doi: 10.1002/ggn2.202500012 (PMC12747557; doi:10.1002/ggn2.202500012)
Supplement: Supplementary file 2 — Supporting file: ggn270014‐sup‐0002‐FigureS1.pdf [file GGN2-6-e00012-s025.pdf]

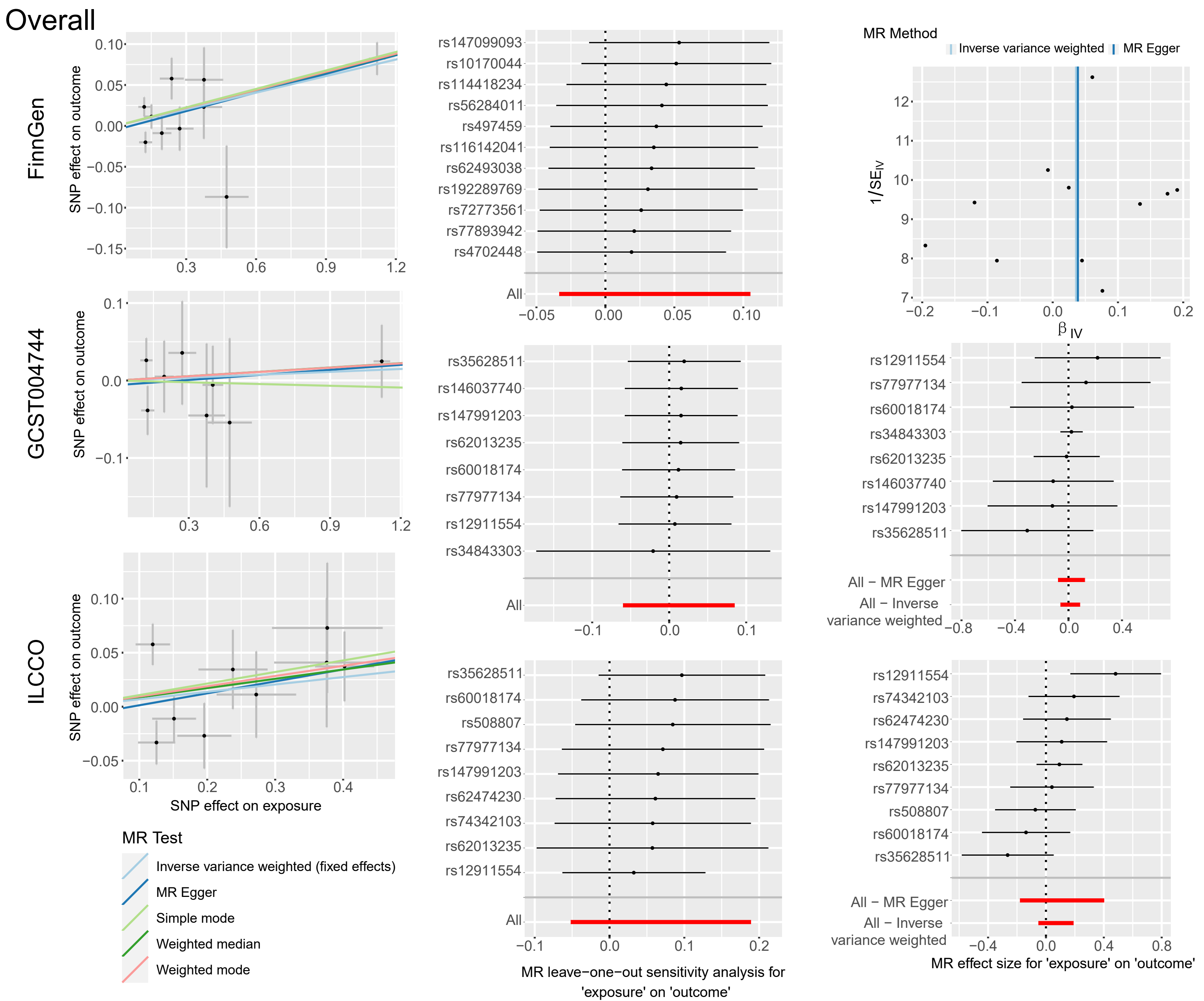

Overall
FinnGen
GCST004744
ILCCO
SNP effect on outcome
SNP effect on exposure
MR Test
Inverse variance weighted (fixed effects)
MR Egger
Simple mode
Weighted median
Weighted mode
rs147099093
rs10170044
rs114418234
rs56284011
rs497459
rs116142041
rs62493038
rs192289769
rs72773561
rs77893942
rs4702448
All
rs35628511
rs146037740
rs147991203
rs62013235
rs60018174
rs77977134
rs12911554
rs34843303
rs508807
rs62474230
rs74342103
MR leave−one−out sensitivity analysis for 'exposure' on 'outcome'
MR Method
Inverse variance weighted
MR Egger
$1/SE_{IV}$
β_{IV}
All − MR Egger
All − Inverse variance weighted
MR effect size for 'exposure' on 'outcome'
