# Supplementary material for: Identification of Cathepsin H and Metabolic Traits as Potential Biomarkers for Lung Cancer by Mendelian Randomization and Single‐Cell Transcriptomics
Source: Adv Genet (Hoboken). 2025 Nov 14;6(4):e00012. doi: 10.1002/ggn2.202500012 (PMC12747557; doi:10.1002/ggn2.202500012)
Supplement: Supplementary file 4 — Supporting file: ggn270014‐sup‐0004‐FigureS3.pdf [file GGN2-6-e00012-s008.pdf]

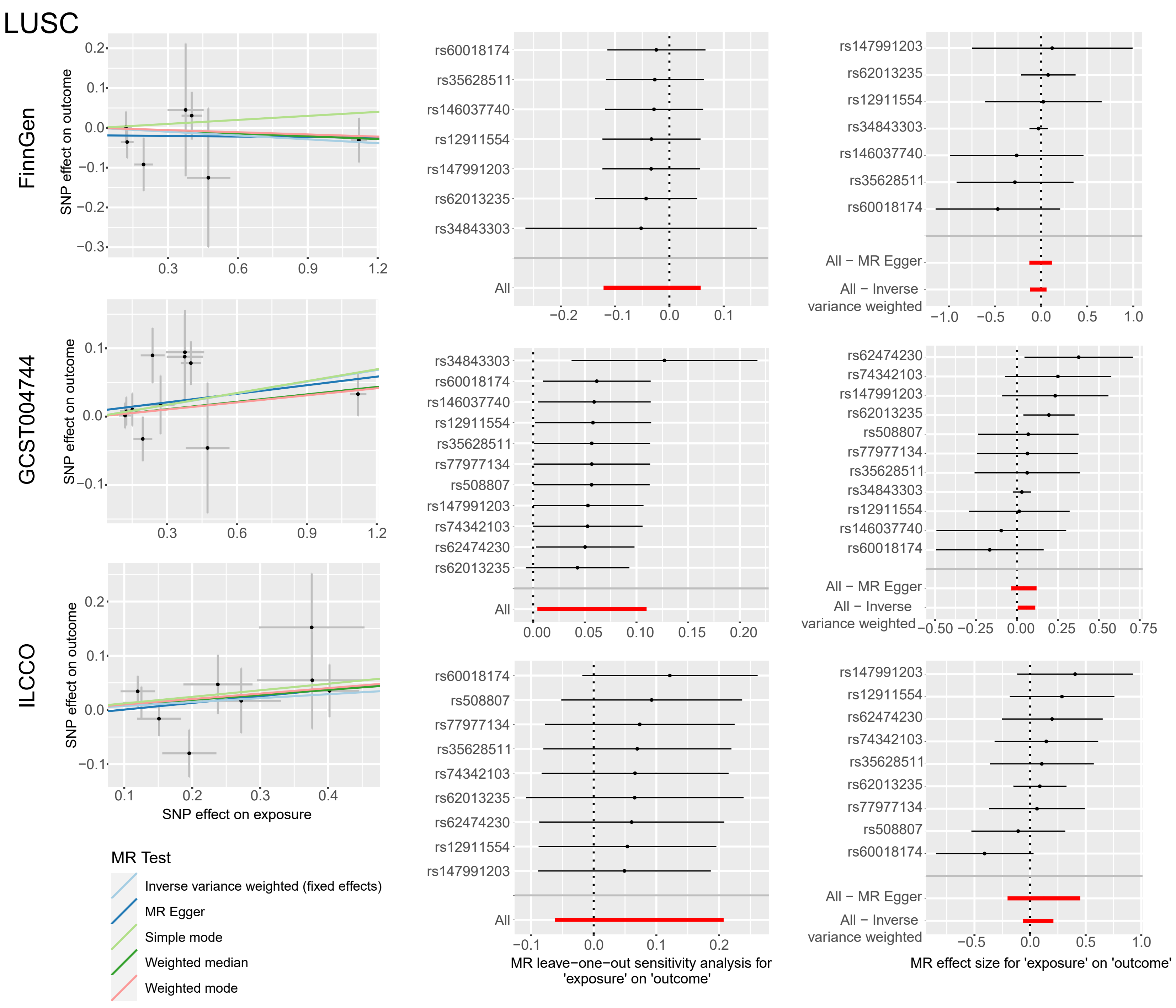

LUSC
FinnGen
GCST004744
ILCCO
SNP effect on outcome
SNP effect on exposure
MR Test
Inverse variance weighted (fixed effects)
MR Egger
Simple mode
Weighted median
Weighted mode
rs60018174
rs35628511
rs146037740
rs12911554
rs147991203
rs62013235
rs34843303
rs77977134
rs508807
rs74342103
rs62474230
All
All − MR Egger
All − Inverse variance weighted
MR leave−one−out sensitivity analysis for 'exposure' on 'outcome'
MR effect size for 'exposure' on 'outcome'
