# Supplementary figures and images for: Identification of Cathepsin H and Metabolic Traits as Potential Biomarkers for Lung Cancer by Mendelian Randomization and Single‐Cell Transcriptomics
Source: Adv Genet (Hoboken). 2025 Nov 14;6(4):e00012. doi: 10.1002/ggn2.202500012 (PMC12747557; doi:10.1002/ggn2.202500012)

# SCLC

## FinnGen

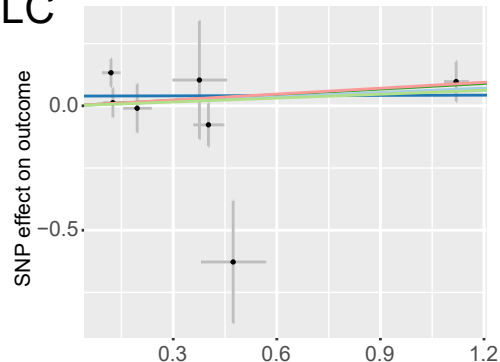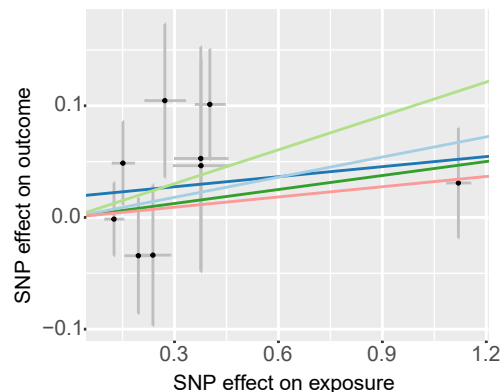

MR Test

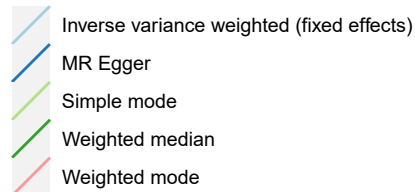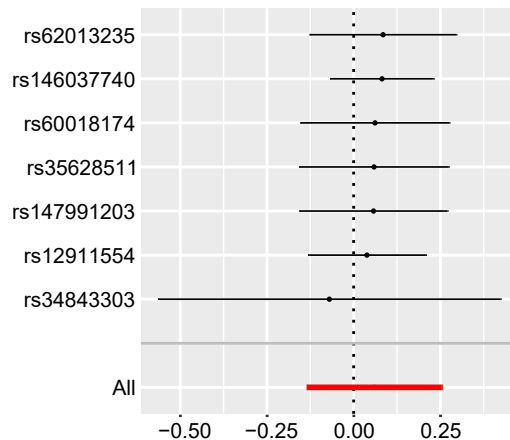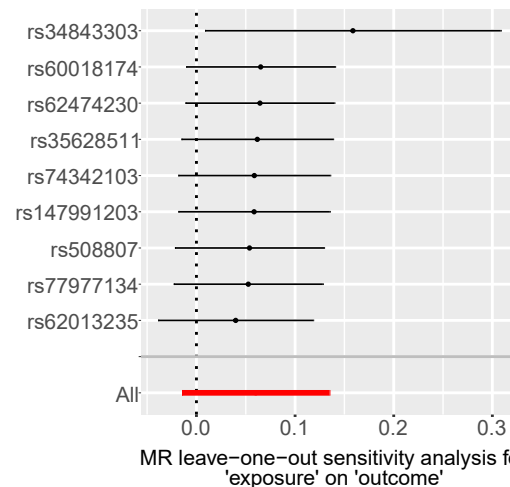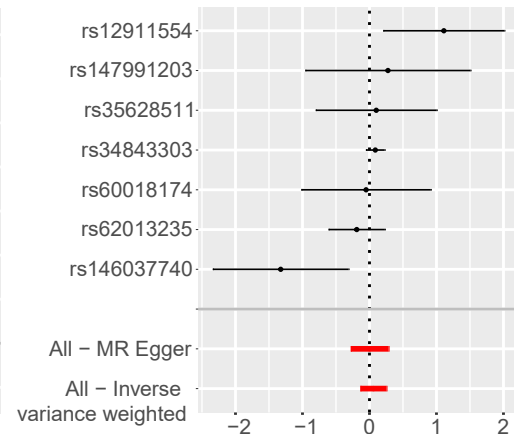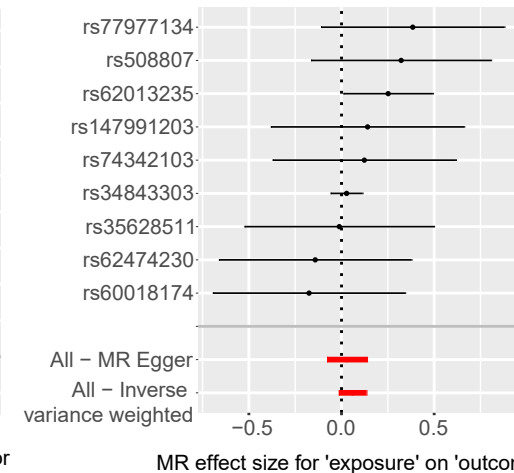

Supplement: Supplementary file 5 — Supporting file: ggn270014‐sup‐0005‐FigureS4.pdf [file GGN2-6-e00012-s004.pdf]

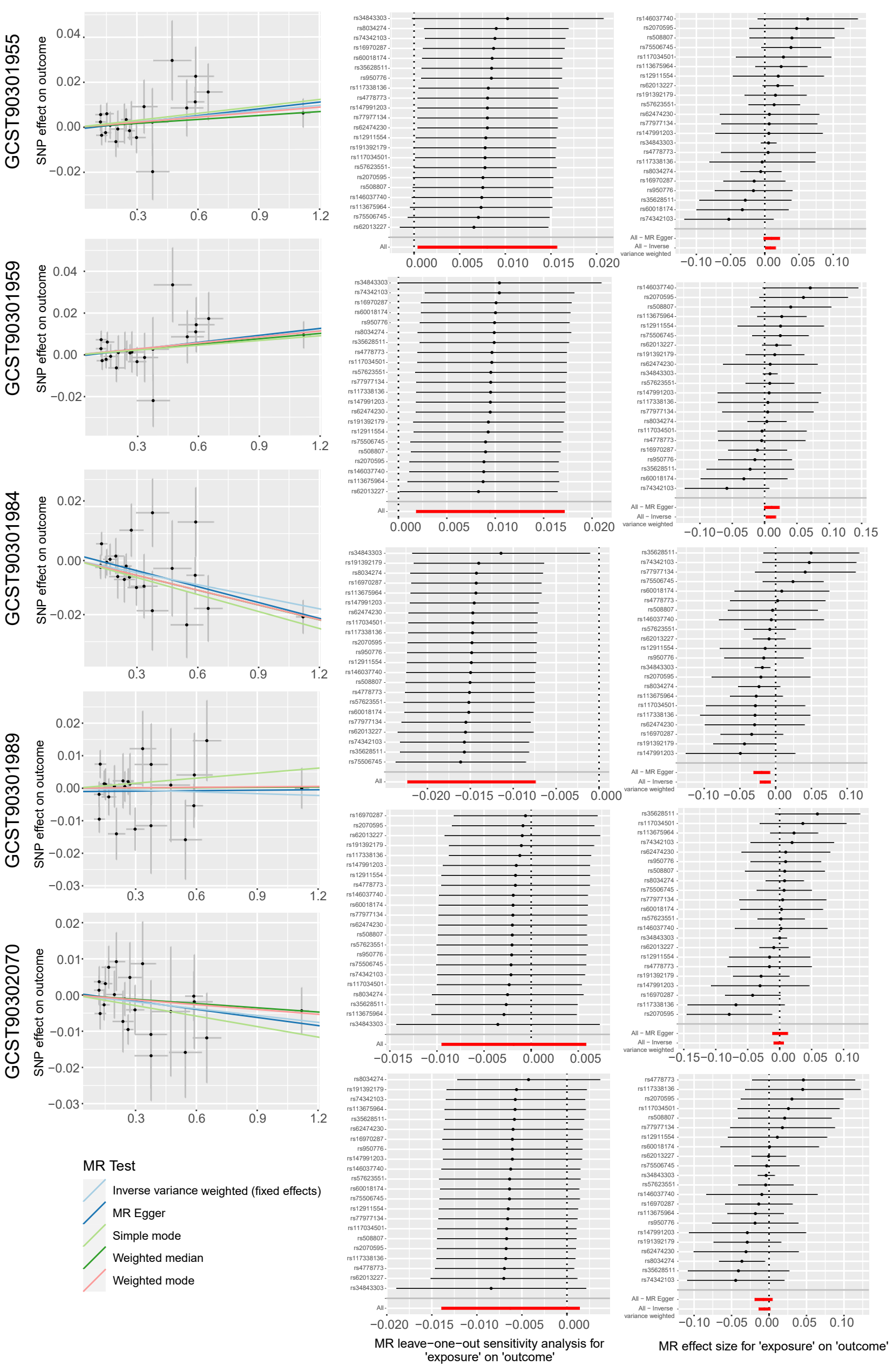

Supplement: Supplementary file 8 — Supporting file: ggn270014‐sup‐0008‐FigureS7.pdf [file GGN2-6-e00012-s013.pdf]

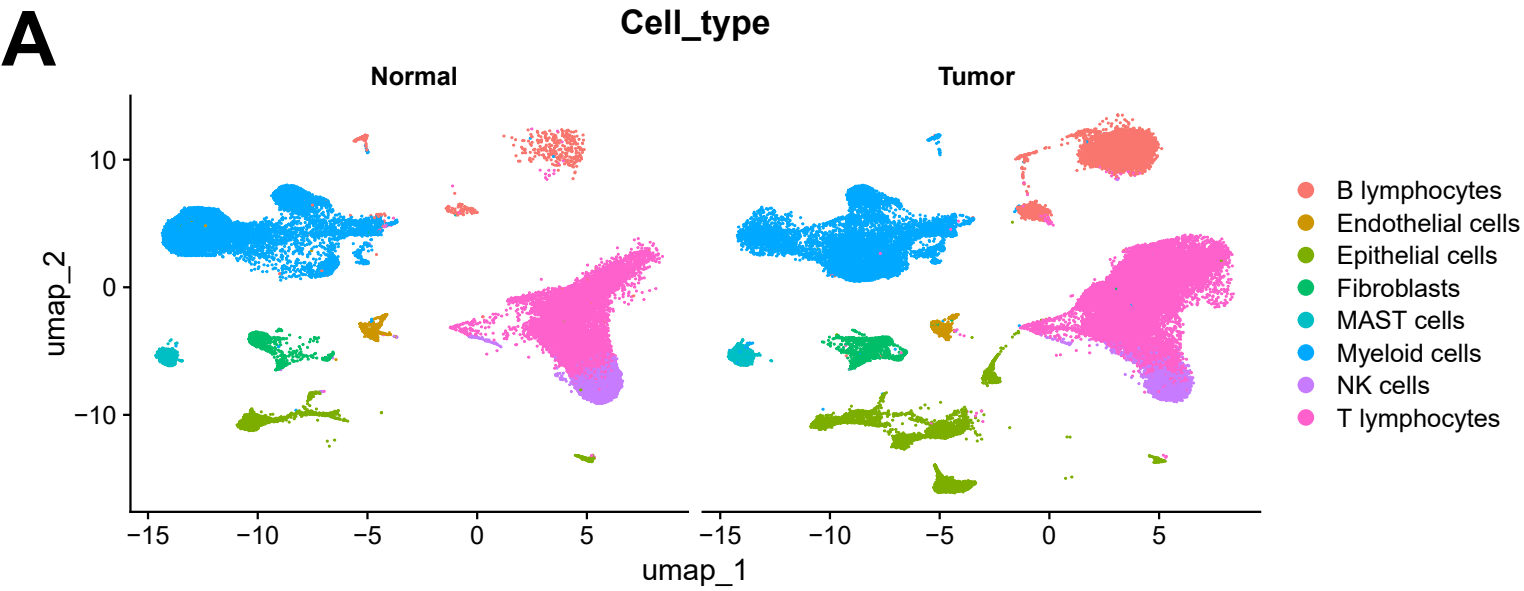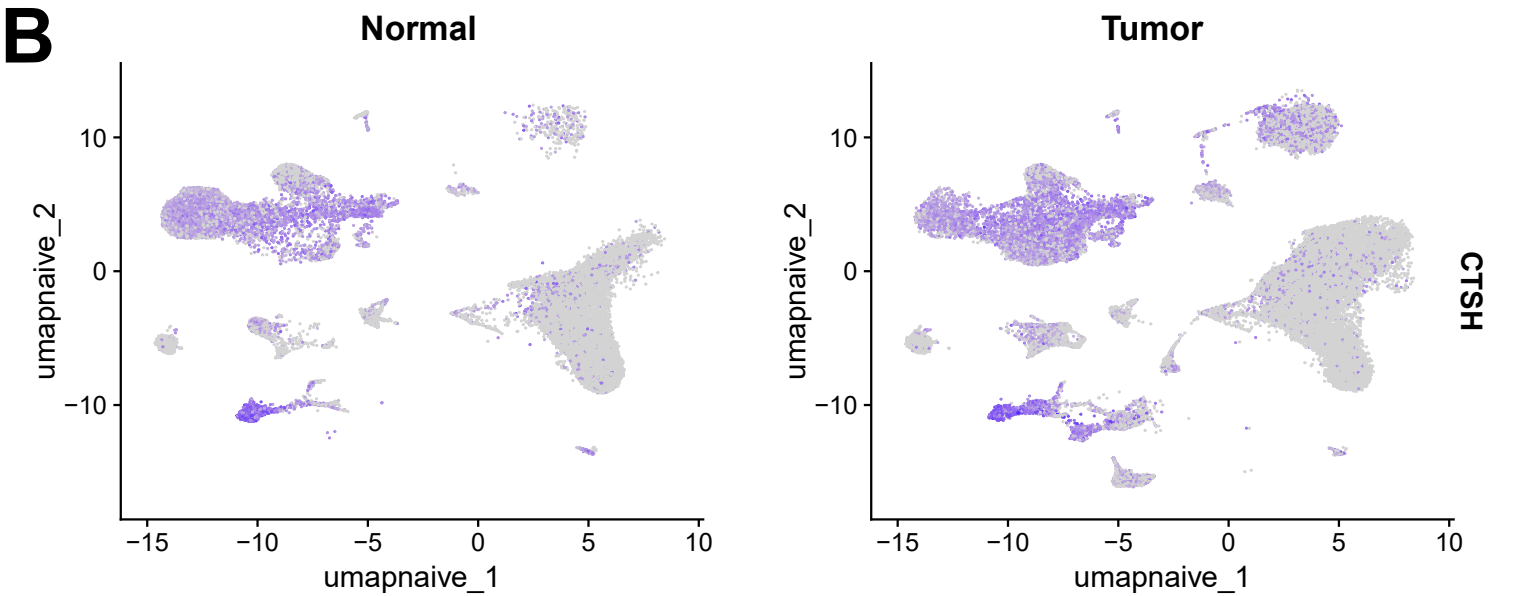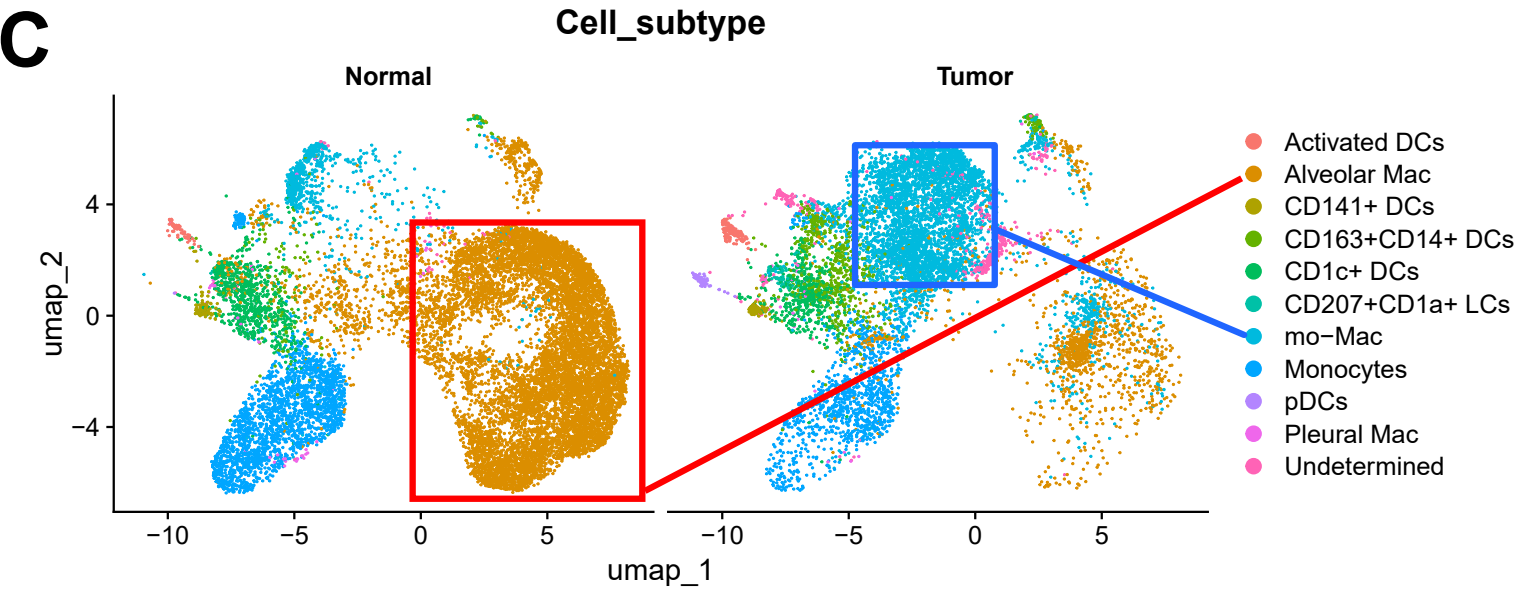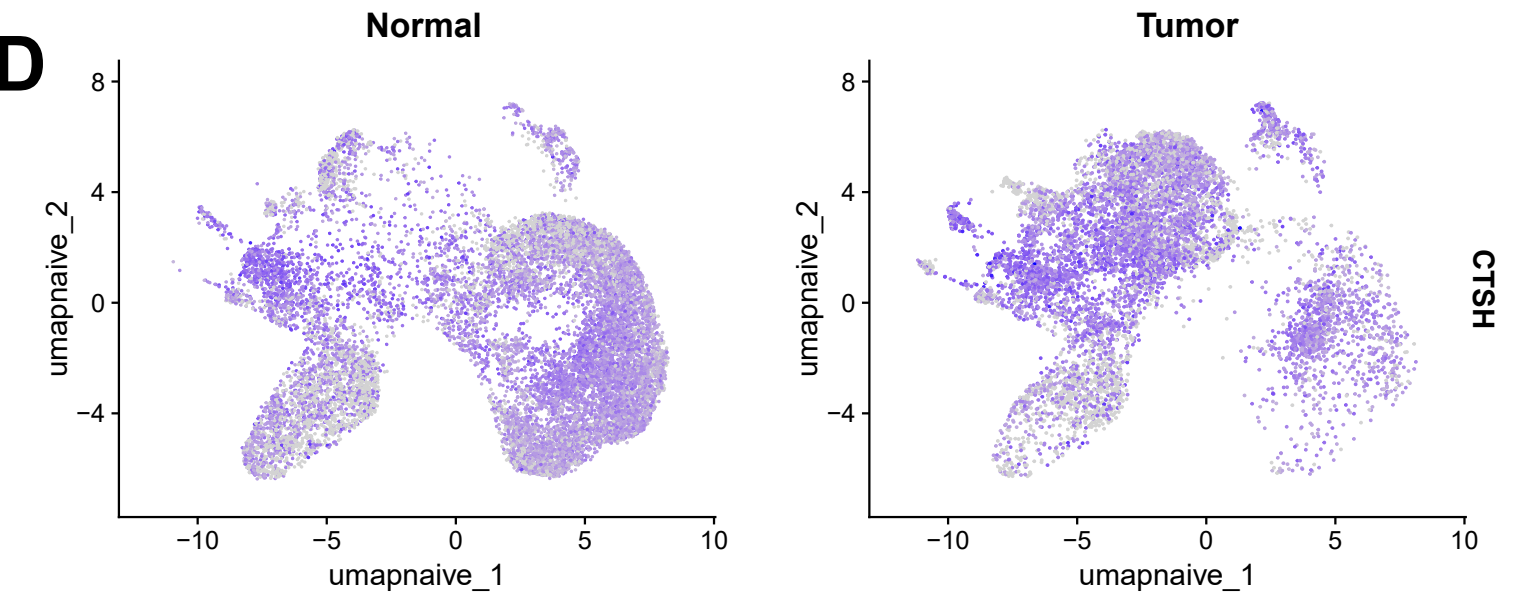

Supplement: Supplementary file 9 — Supporting file: ggn270014‐sup‐0009‐FigureS8.pdf [file GGN2-6-e00012-s015.pdf]

### Activated DCs

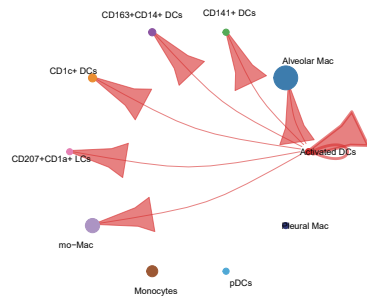

### CD141+ DCs

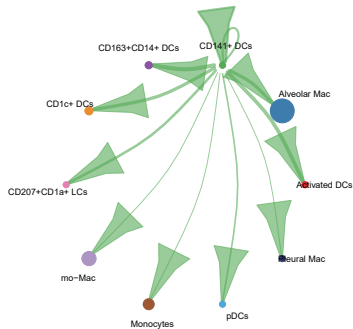

### CD163+CD14+ DCs

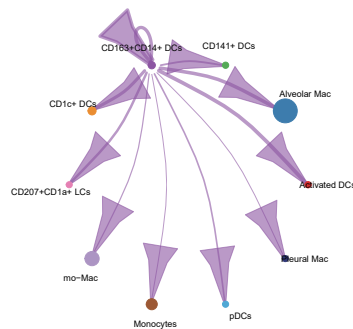

### CD1c+ DCs

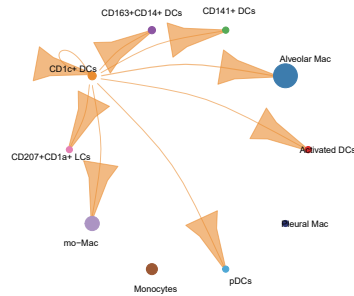

### CD207+CD1a+ LCs

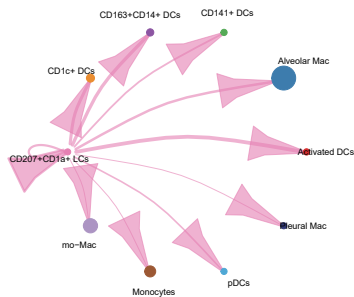

### Monocytes

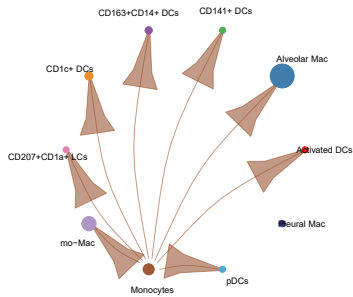

### pDCs

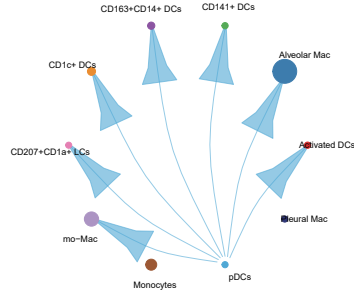

### Pleural Mac

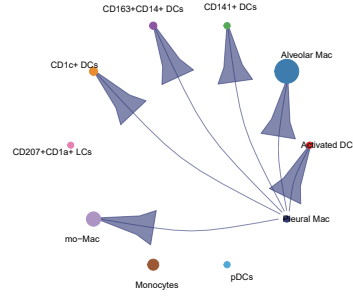

Supplement: Supplementary file 10 — Supporting file: ggn270014‐sup‐0010‐FigureS9.pdf [file GGN2-6-e00012-s010.pdf]

## Contribution of each L-R pair

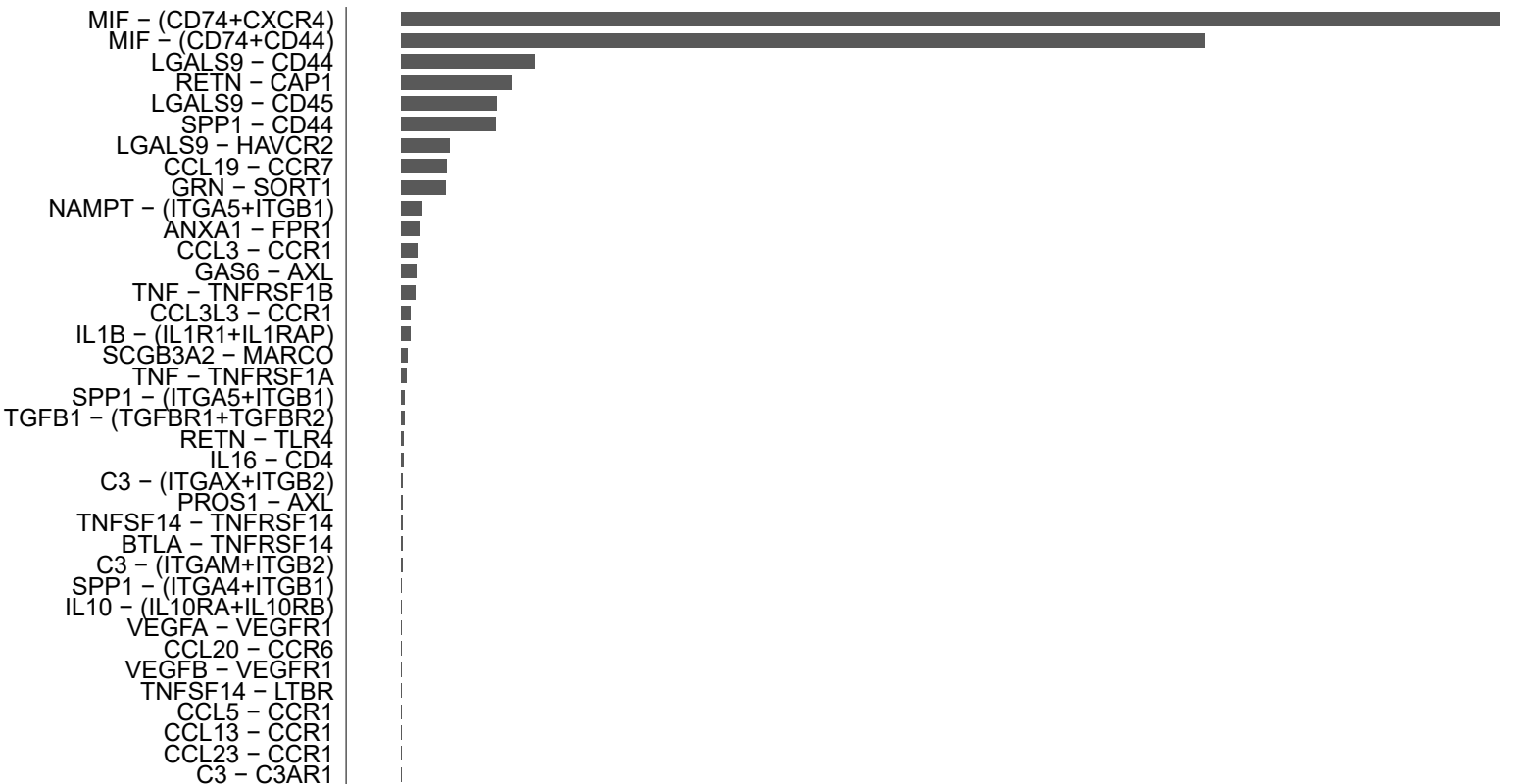

Relative contribution

Supplement: Supplementary file 11 — Supporting file: ggn270014‐sup‐0011‐FigureS10.pdf [file GGN2-6-e00012-s016.pdf]

A

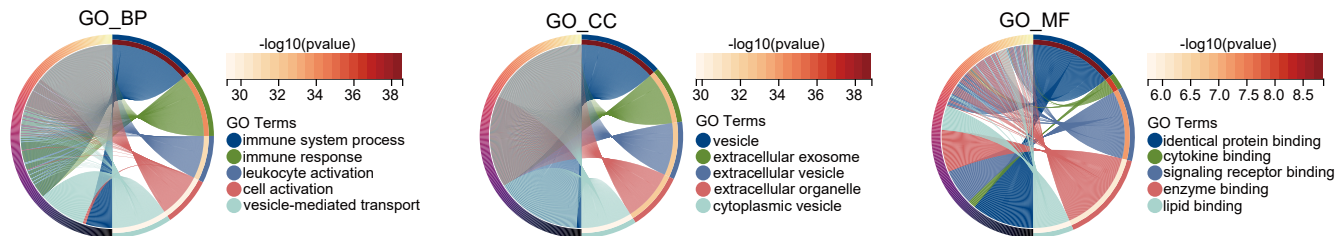

B

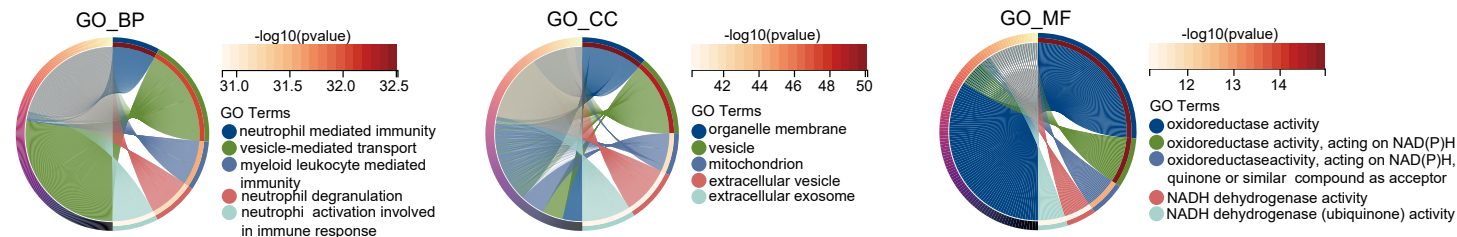

C

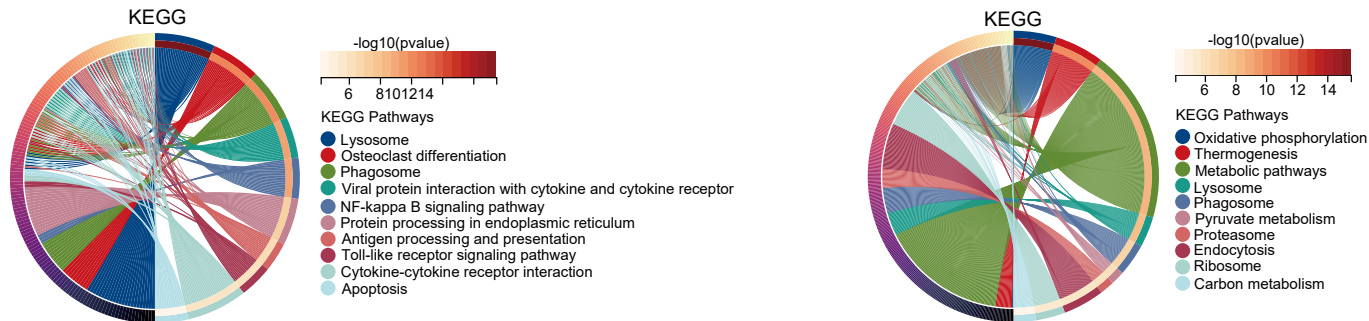

mo-Mac

Alveolar Mac

Supplement: Supplementary file 12 — Supporting file: ggn270014‐sup‐0012‐FigureS11.pdf [file GGN2-6-e00012-s020.pdf]

# Cell\_subtype

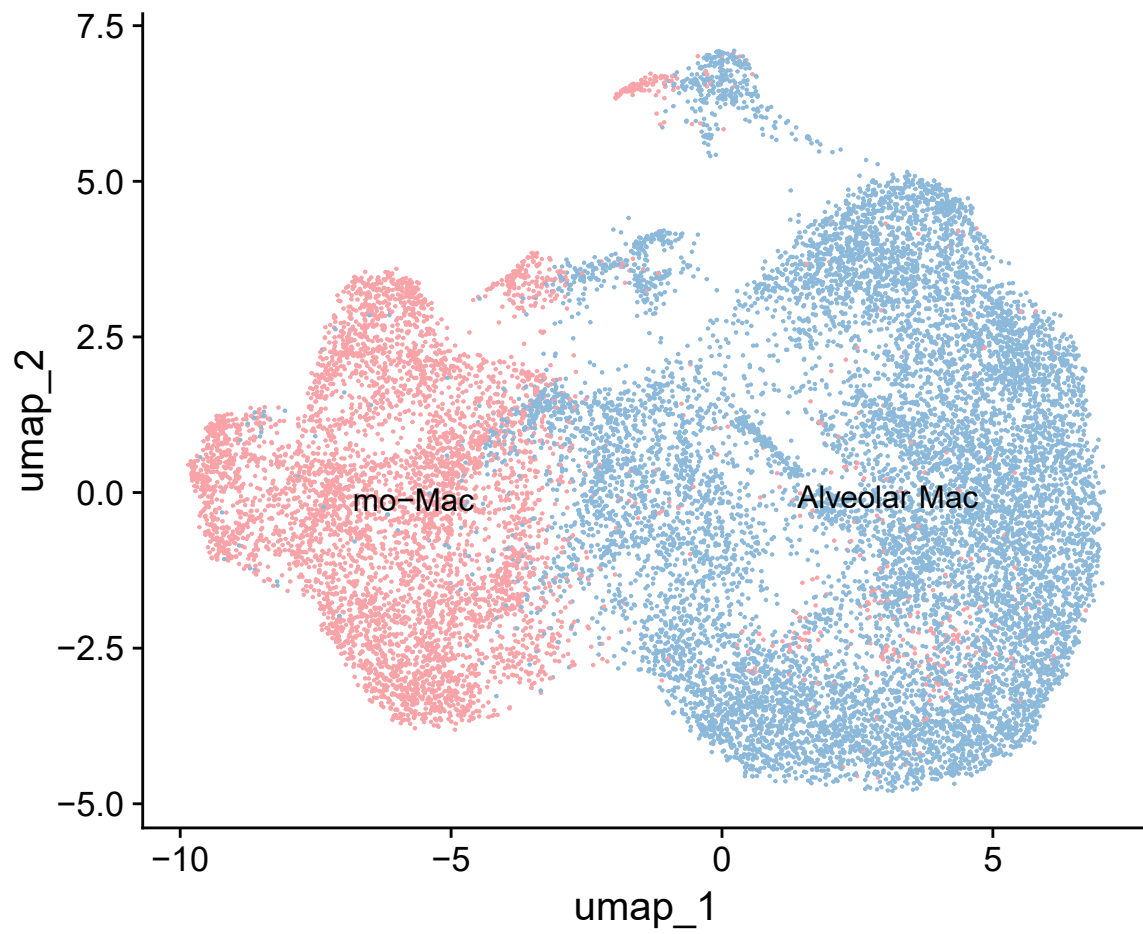

## CTSH

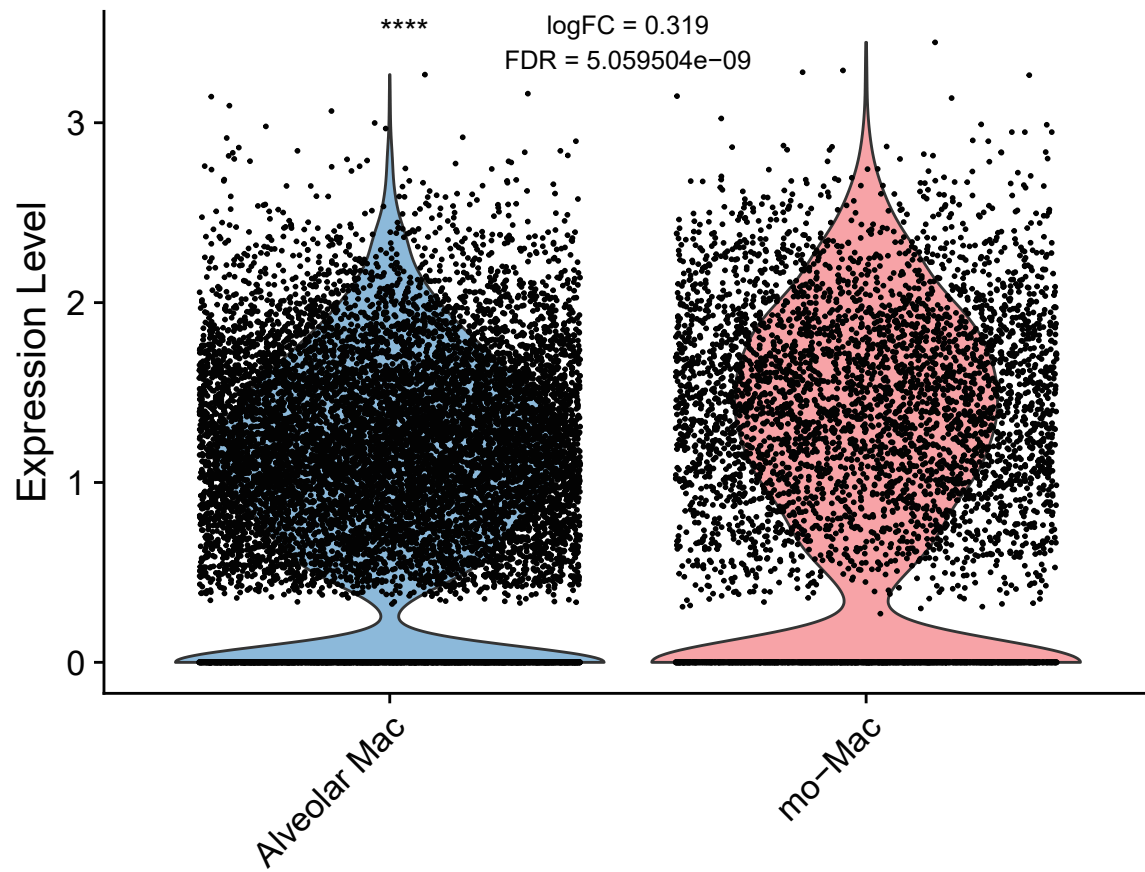

Supplement: Supplementary file 14 — Supporting file: ggn270014‐sup‐0014‐FigureS13.pdf [file GGN2-6-e00012-s014.pdf]
