# Supplementary material for: Identification of Cathepsin H and Metabolic Traits as Potential Biomarkers for Lung Cancer by Mendelian Randomization and Single‐Cell Transcriptomics
Source: Adv Genet (Hoboken). 2025 Nov 14;6(4):e00012. doi: 10.1002/ggn2.202500012 (PMC12747557; doi:10.1002/ggn2.202500012)
Supplement: Supplementary file 6 — Supporting file: ggn270014‐sup‐0006‐FigureS5.pdf [file GGN2-6-e00012-s023.pdf]

GCST004744

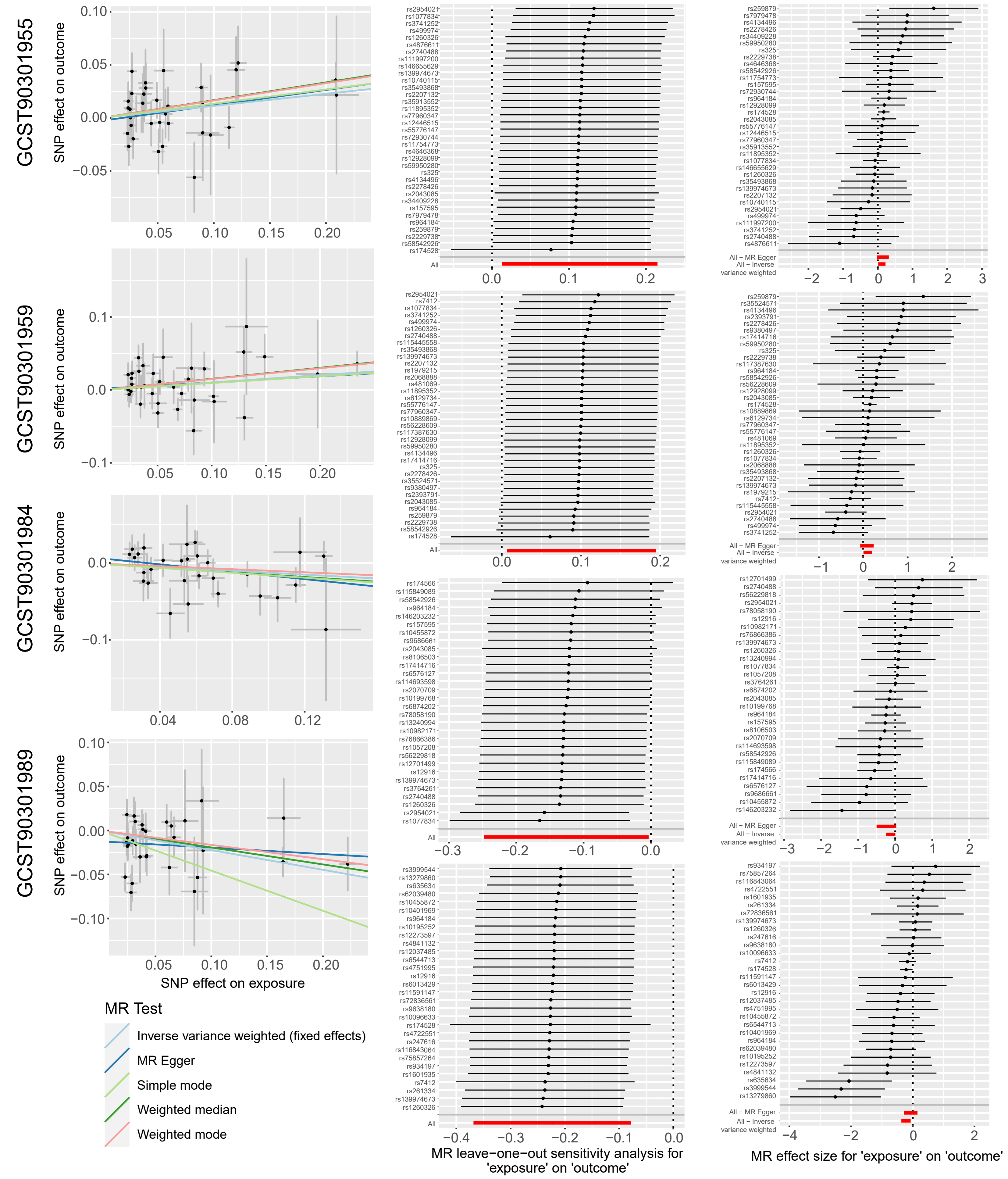

GCST90301955
GCST90301959
GCST90301984
GCST90301989
SNP effect on outcome
SNP effect on exposure
MR Test
Inverse variance weighted (fixed effects)
MR Egger
Simple mode
Weighted median
Weighted mode
MR leave−one−out sensitivity analysis for 'exposure' on 'outcome'
MR effect size for 'exposure' on 'outcome'
All − MR Egger
All − Inverse variance weighted
All
