# Supplementary material for: Identification of Cathepsin H and Metabolic Traits as Potential Biomarkers for Lung Cancer by Mendelian Randomization and Single‐Cell Transcriptomics
Source: Adv Genet (Hoboken). 2025 Nov 14;6(4):e00012. doi: 10.1002/ggn2.202500012 (PMC12747557; doi:10.1002/ggn2.202500012)
Supplement: Supplementary file 13 — Supporting file: ggn270014‐sup‐0013‐FigureS12.pdf [file GGN2-6-e00012-s021.pdf]

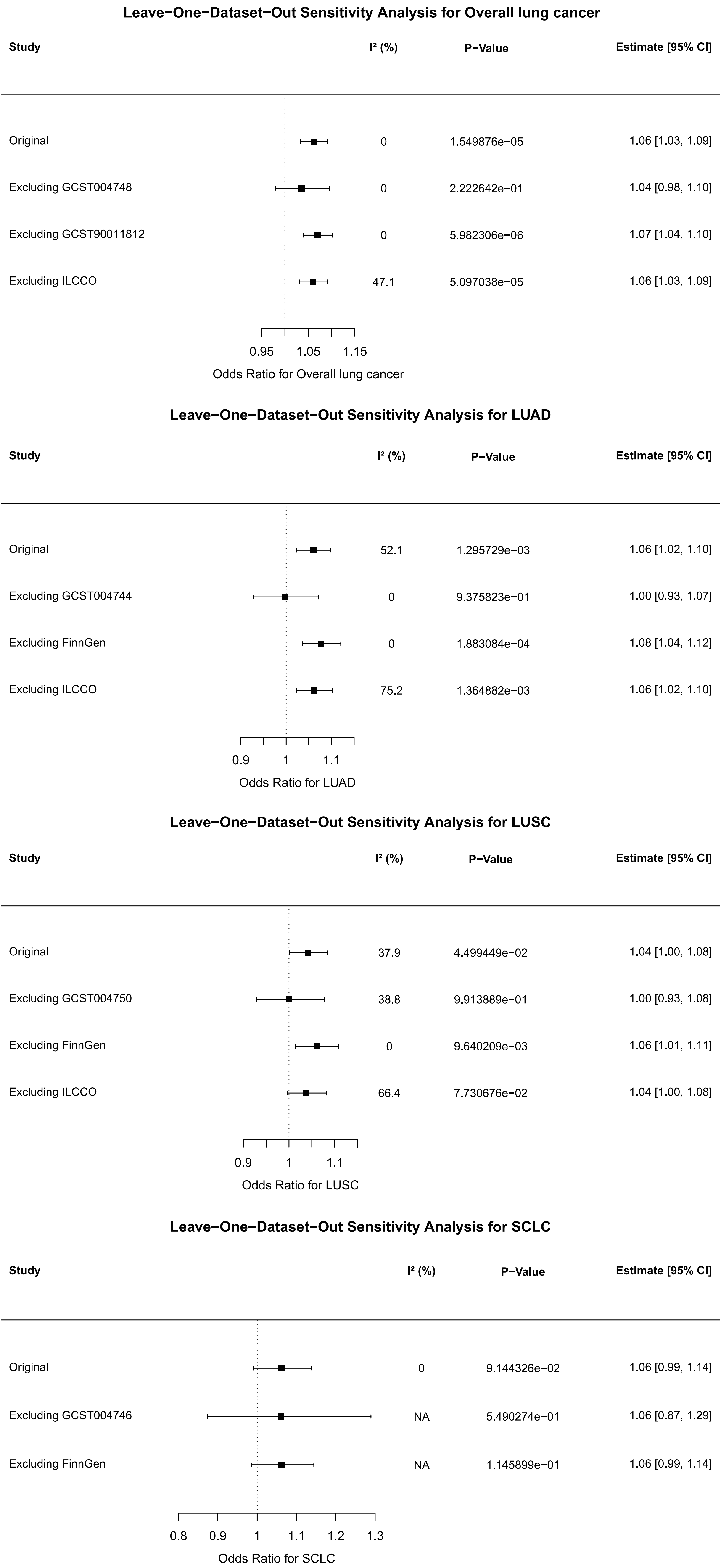
Leave−One−Dataset−Out Sensitivity Analysis for Overall lung cancer
Study
I² (%)
P−Value
Estimate [95% CI]
Original
0
1.549876e−05
1.06 [1.03, 1.09]
Excluding GCST004748
0
2.222642e−01
1.04 [0.98, 1.10]
Excluding GCST90011812
0
5.982306e−06
1.07 [1.04, 1.10]
Excluding ILCCO
47.1
5.097038e−05
1.06 [1.03, 1.09]
0.95
1.05
1.15
Odds Ratio for Overall lung cancer
Leave−One−Dataset−Out Sensitivity Analysis for LUAD
Study
I² (%)
P−Value
Estimate [95% CI]
Original
52.1
1.295729e−03
1.06 [1.02, 1.10]
Excluding GCST004744
0
9.375823e−01
1.00 [0.93, 1.07]
Excluding FinnGen
0
1.883084e−04
1.08 [1.04, 1.12]
Excluding ILCCO
75.2
1.364882e−03
1.06 [1.02, 1.10]
0.9
1
1.1
Odds Ratio for LUAD
Leave−One−Dataset−Out Sensitivity Analysis for LUSC
Study
I² (%)
P−Value
Estimate [95% CI]
Original
37.9
4.499449e−02
1.04 [1.00, 1.08]
Excluding GCST004750
38.8
9.913889e−01
1.00 [0.93, 1.08]
Excluding FinnGen
0
9.640209e−03
1.06 [1.01, 1.11]
Excluding ILCCO
66.4
7.730676e−02
1.04 [1.00, 1.08]
0.9
1
1.1
Odds Ratio for LUSC
Leave−One−Dataset−Out Sensitivity Analysis for SCLC
Study
I² (%)
P−Value
Estimate [95% CI]
Original
0
9.144326e−02
1.06 [0.99, 1.14]
Excluding GCST004746
NA
5.490274e−01
1.06 [0.87, 1.29]
Excluding FinnGen
NA
1.145899e−01
1.06 [0.99, 1.14]
0.8
0.9
1
1.1
1.2
1.3
Odds Ratio for SCLC
