# Supplementary material for: Identification of Cathepsin H and Metabolic Traits as Potential Biomarkers for Lung Cancer by Mendelian Randomization and Single‐Cell Transcriptomics
Source: Adv Genet (Hoboken). 2025 Nov 14;6(4):e00012. doi: 10.1002/ggn2.202500012 (PMC12747557; doi:10.1002/ggn2.202500012)
Supplement: Supplementary file 26 — Supplementary Information: Record of Transparent Peer Review [file GGN2-6-e00012-s019.docx]

Record of Transparent Peer Review

Identification of Cathepsin H and Metabolic Traits as Potential Biomarkers for Lung Cancer by Mendelian Randomization and Single-Cell Transcriptomics

Data submitted: 08-Apr-2025

1^st^ Editorial Decision: 19-May-2025

1^st^ Revision Received: 10-Sep-2025

2^nd^ Editorial Decision: 07-Oct-2025

2^nd^ Revision Received: 18-Oct-2025

3^rd^ Editorial Decision: 02-Nov-2025

3^rd^ Revision Received: 02-Nov-2025

Accepted: 03-Nov-2025

**Revision with responses: 10-Sep-2025 (1^st^ Revision )**

**Reviewer #2:**

**Comments to the Author:**

1. The authors analyzed lung cancer mortality rates attributable to fasting hyperglycemia, but the longitudinal patterns of lung cancer's incidence trends associated with fasting hyperglycemia were also discussed.

**Author response and action taken:**

We thank the reviewer for pointing out this inconsistency between incidence and mortality. In the revised Results section (3.1), we clarified that our primary analysis concerned mortality attributable to fasting hyperglycemia. The incidence trends presented in Figure 2A were described separately as background epidemiological context, not as a causal consequence of hyperglycemia.

**Comments to the Author:**

2. Is CTSH a significant pathogenic factor for lung cancer? Has preliminary consensus been reached regarding the validity of its data nodes?

**Author response and action taken:**

We thank the reviewer for this key question. Current GWAS and multi-database MR analyses indeed support an association between CTSH and lung cancer risk. However, we agree that there is no definitive mechanistic or experimental consensus at present. Accordingly, we have added explanatory text in the Discussion.

**Comments to the Author:**

3. In Line-115, CTSH involved in cellular metabolic regulation including AA and lipid，but glycometabolism, which is the key fator in this article, was not mentioned.

**Author response and action taken:**

We appreciate this useful suggestion. Indeed, CTSH has also been implicated in glycometabolic regulation, especially under hyperglycemic conditions. We have revised the Introduction section.

**Comments to the Author:**

4. The relationship between CTSH and fasting hyperglycemia was not discussed in depth in the manuscript, and the possible relationship between CTSH, fasting hyperglycemia, and lung cancer was also not discussed. Why did authors choose lung cancer under blood sugar instead of other chronic diseases?

**Author response and action taken:**

We agree with the reviewer that this linkage deserved further elaboration. We expanded the discussion section with some additional content.

**Comments to the Author:**

5. In Line-457, pDCs should be no significant difference ( p≥0.05) shown by Figure 5f.

**Author response and action taken:**

We thank the reviewer for catching this discrepancy. The Results text and figure labels were inconsistent. We have corrected the Results section 3.6.

**Reviewer #3:**

**Comments to the Author:**

1. Major Concern 1: Causality Strength and Dataset Inconsistency

While the authors conclude that elevated CTSH is causally linked to an increased risk of lung cancer, particularly adenocarcinoma, the strength of the association appears marginal. The odds ratio from meta-analysis is modest (OR ≈ 1.06), and the findings are inconsistent across datasets: the McKay dataset shows significant results, whereas FinnGen and ILCCO do not. This heterogeneity raises concerns about the robustness of the causal inference. The authors should present a more critical discussion of the limitations of weak effect sizes and explore the potential role of residual confounding or horizontal pleiotropy despite the use of MR-PRESSO and sensitivity tests.

**Author response and action taken:**

We thank the reviewer for pointing out this important issue. We fully agree that the association strength is modest and heterogeneous across datasets. In the revised Discussion, we expanded the limitations to clearly state:

(1) The pooled meta-analysis yielded a borderline OR≈1.06, which should be interpreted as a modest effect size.
(2) The inconsistency across datasets (McKay-positive vs. FinnGen/ILCCO-negative) reflects population heterogeneity, sample size differences, and possible residual confounding.
(3) Although we conducted MR-PRESSO and sensitivity tests (Cochran’s Q, MR-Egger intercept), these methods cannot fully rule out pleiotropy or hidden confounding.

Moreover, we have explicitly re-phrased our conclusions to emphasize that findings suggest a possible but modest causative role for CTSH, requiring replication in larger harmonized cohorts.

**Comments to the Author:**

2. Major Concern 2: Inconsistency Between Transcriptomic and Proteomic CTSH Expression

There is a notable discrepancy between CTSH mRNA and protein expression in tumors, which is not clearly communicated or reconciled in the manuscript. According to the Results section, CTSH expression is lower at the mRNA level in tumors (TCGA data) but higher at the protein level (HPA immunohistochemistry). However, the Abstract states that "validation confirmed CTSH overexpression in tumors," without specifying whether this refers to RNA or protein data. This leads to a misleading interpretation, as transcriptomic and proteomic results point in opposite directions.

Moreover, the HPA analysis is based on publicly available tissue staining images and does not constitute an experimental validation. The authors should revise the Abstract to clearly state that only protein-level data suggest elevated CTSH, and explicitly acknowledge the discordance with transcriptomic data. If CTSH expression is a central pillar of the paper's conclusions, further validation via qPCR or immunoblotting in matched samples would substantially strengthen the claim.

**Author response and action taken:**

We thank the reviewer for this very valuable comment. We agree that transcriptomic (TCGA) and proteomic (HPA) results showed discordant patterns. We have made the following revisions:

In the Discussion section, we expanded to explain possible biological reasons for this discrepancy, including post-transcriptional regulation, altered protein stability, or technical differences between datasets. We also acknowledged that the HPA dataset, while informative, does not constitute direct experimental validation. To address this limitation, we have added PCR and WB for further independent validation in matched tumor/normal samples, which revealed opposing expression trends of CTSH at the RNA and protein levels (in Fig.6 M-N ).

**Comments to the Author:**

3. Minor Concern 1: Biological Relevance of Mediation Analysis

The mediation effects of DHA (22:6) and omega-3 fatty acids in the CTSH-lung adenocarcinoma pathway are statistically significant but biologically limited (1.1-1.2%). The authors should clarify the biological meaning of such low mediation proportions and avoid overstating the relevance of the metabolic mediation pathway. A brief discussion on whether these effects are likely to be mechanistically relevant in vivo would be helpful.

**Author response and action taken:**

We appreciate this constructive suggestion. We agree with the reviewer that the mediation proportions, although statistically significant, represent relatively small effect sizes. To address this, we have revised the Discussion to explicitly state that these modest mediation effects (~1–2%) may have limited biological relevance and should be interpreted with caution. We now describe them as hypothesis-generating findings rather than conclusive evidence of a major metabolic pathway.

Reviewer #4:

**Comments to the Author:**

1. Several CTSH-cancer associations (e.g., from Rshkin and ILCCO) were non-significant. Although the meta-analysis pooled positive and null results, the manuscript should more thoroughly discuss possible sources of heterogeneity (e.g., case definition, population structure, instrument strength) and sensitivity of conclusions to inclusion/exclusion of cohorts.

**Author response and action taken:**

We thank the reviewer for this important suggestion. We agree that heterogeneity across cohorts is a key limitation. In the revised Discussion (Lines xxx–xxx), we have expanded the interpretation to explicitly acknowledge that:

(1)Non-significant associations in ILCCO and Rashkin may reflect differences in case definition, population composition, and instrument strength.

(2)Although we did not perform an additional “leave-one-cohort out” meta-analysis, the current design already included heterogeneity tests (Cochran’s Q) and pleiotropy assessments (MR-PRESSO and MR-Egger intercept), all of which indicated no major violations.

(3)Furthermore, our original leave-one-SNP analyses in each cohort showed consistent directional effects, supporting robustness of individual dataset results.

Accordingly, we have revised the Discussion to emphasize that while the pooled results (meta-analysis OR≈1.06) provide directionally consistent evidence, the findings are modest, preliminary, and subject to heterogeneity across cohorts, and thus should be interpreted with caution. Larger harmonized GWAS datasets will be required to further confirm robustness in future work.

**Comments to the Author:**

2. The estimated mediation proportions (~1-1.2%) for DHA and omega-3 are modest. The authors should comment on whether such small indirect effects are biologically meaningful, and discuss statistical power/precision of Sobel estimates given instrument strength.

**Author response and action taken:**

We greatly appreciate this insightful comment. As also noted by Reviewer #3 (Minor Concern 3), the mediation proportions for DHA (22:6) and omega-3 fatty acids were indeed small (~1-1.2%), and in response we had already revised the Discussion to clarify that such effects suggest limited biological contribution and should be regarded as hypothesis-generating rather than conclusive.

Following this additional comment, we have further expanded this section of the Discussion to explicitly address the issue of statistical power and Sobel test precision. Specifically, we now state that although instrumental SNPs had adequate strength (F-statistics >10), the Sobel method is conservative and has limited precision for detecting small indirect effects, which may partly explain the modest mediation values observed. We also emphasize that larger GWAS datasets will be required to robustly quantify mediation.

**Comments to the Author:**

3. The paper applies different significance thresholds (p<5×10⁻⁶ for CTSH instruments; p<5×10⁻⁸ for metabolites). Please justify these choices explicitly and describe how FDR correction was applied across the multiple MR and mediation tests to control type I error.

**Author response and action taken:**

We thank the reviewer for the opportunity to clarify this point. Due to the relatively limited number of genome-wide significant SNPs available for CTSH, we adopted a relaxed threshold of p < 5×10⁻⁶ for instrumental SNP selection, an approach consistent with previous Mendelian randomization studies of circulating proteins where fewer variants are available. By contrast, for metabolites we applied the conventional genome-wide significance threshold of p < 5×10⁻⁸, since sufficient genome-wide significant instruments exist.To control type I error, we explicitly applied false discovery rate (FDR q < 0.05) correction within each analytical framework. Specifically, FDR was applied across: (1) The MR analyses of 233 metabolites; (2) The two-step mediation analyses. This ensured that multiple testing burden was appropriately addressed within each analytic domain.

**Comments to the Author:**

4. The finding that DHA and omega-3 levels increase lung adenocarcinoma risk runs counter to most literature describing anti-inflammatory/anti-tumor effects. The Discussion notes this but does not propose mechanistic explanations. The authors should expand on possible dietary, enzymatic, or context-dependent effects that might reverse expected directions, or consider residual pleiotropy.

**Author response and action taken:**

We thank the reviewer for this important comment. We agree that our observation that DHA and omega-3 fatty acids appear to increase lung adenocarcinoma risk contrasts with the conventional view of these nutrients as anti-inflammatory and anti-tumorigenic. In response, we have expanded the Discussion (Lines xxx–xxx) to provide potential explanations:

(1) High dietary intake or excessive supplementation of omega-3 may exert paradoxical effects.
(2) Omega-3 fatty acids can be metabolized into diverse lipid mediators; while some have anti-inflammatory properties, others (e.g. pro-resolving lipid mediators, certain eicosanoids) may be context-dependent and could facilitate tumor progression in certain settings.
(3) Inflammatory and metabolic conditions in the tumor microenvironment may modulate the effects of omega-3s, reversing their anticipated anti-tumor activity.
(4) Despite sensitivity analyses, the possibility of residual pleiotropy cannot be entirely excluded and may partly contribute to this unexpected association.

We also emphasize that these findings should be considered preliminary and hypothesis-generating, highlighting the need for mechanistic and experimental studies to corroborate the directions observed in genetic analyses.

**Comments to the Author:**

5 .Minor Points:

(1)Please specify why a 25% cutoff and min.cell =10 were chosen in CellChat, and whether results are robust to different thresholds.

**Author response and action taken:**

we have now clarified in the Methods section that the choice of a 25% cutoff and min.cell = 10 follows the default recommendations of the CellChat package and is widely adopted in prior studies to filter low‑quality signals. While we did not perform additional re‑analyses with alternative thresholds in this study, it is expected that the overall interaction trends would remain consistent given the robustness of the default pipeline. We have acknowledged this as a limitation and noted that further testing of different parameter settings will be addressed in future work.

**Comments to the Author:**

5. Minor Points:

(2)The manuscript references Supplementary Figures 11 and Table 9, but these appear out of sequence. Please verify numbering and that each supplement is cited at the correct point in the text.

**Author response and action taken:**

We carefully re-checked the Supplementary materials and corrected the numbering of **Supplementary Figure 11** and **Table 9**. They are now cited in the correct sequence within the Results section.

**Revision with responses: 18-Oct-2025 ( 2^nd^ Revision )**

Reviewer Comments

**Comments to the Author**

Which aspects of scholarly presentation require improvement (if any)?

Reviewer #2:

*Supporting Information

Reviewer #3:

*Clarity

*Writing style

**Author response and action taken:**

Thank you for your valuable feedback on improving scholarly presentation. For Reviewer #2’s comment on “Supporting Information”, we have thoroughly reviewed and revised Supplementary Figures 1-6, adding details on sample sizes, statistical methods, p-values, and data presentation to ensure completeness and compliance with journal standards. For Reviewer #3’s suggestions on “Clarity” and “Writing Style”, we engaged a native English-speaking professional to polish the manuscript, simplifying sentence structures and improving paragraph flow to enhance readability and clarity. The revised manuscript and supporting materials have been uploaded via the journal’s online system, adhering to the Manuscript Preparation Checklist.

Reviewer #2:

**Comments to the Author**

I have no further comments to provide.

**Author response:**

Thank you for reviewing our manuscript and providing valuable feedback. We greatly appreciate your confirmation that no further comments are needed.

Reviewer #3:

**Comments to the Author**

1.Effect size & heterogeneity. The MR signals look directionally consistent but tiny (~1.05-1.07) and a bit wobbly across datasets—please add a per-cohort forest plot + leave-one-dataset-out, and tone down the Abstract/Discussion so readers don't overread a small, heterogeneous effect.

**Author response and action taken:**

We thank the reviewer for the helpful suggestion.

Regarding the suggestion to add a per-cohort forest plot, we would like to clarify that Figure 3 already provides per-cohort (dataset) forest plots, showing the individual dataset estimates (GCST004744, FinnGen, ILCCO) along with fixed-effect combined ORs, 95% CIs, weights, and heterogeneity (I²). We have updated the figure legend to explicitly label these as 'per-dataset (cohort) forest plots' and highlighted the cohort-specific contributions, reflecting differences in sample sizes and populations across datasets. This revision addresses the request without the need for additional figures. (**lines 1105-1114**)

For the suggestion to add a leave-one-dataset-out analysis, we have conducted this analysis using Supplementary Table 3 data, implementing the leave-one-dataset-out sensitivity analysis with R's meta package (using the fixed-effect IVW model). We have generated a comprehensive forest plot for each outcome (Overall lung cancer, LUAD, LUSC, SCLC) in **Supplementary Figure 12**, which integrates the original meta-analysis results (OR, 95% CI, I², and P-values) along with the results from excluding one dataset at a time. The quantitative results are presented in **Supplementary Table 10**. The analysis showed that the effect direction was consistent (OR ≈ 1.05-1.07), but excluding certain studies significantly reduced I², suggesting that this study was a major source of heterogeneity. For SCLC, due to only two datasets, I² was not available after exclusion. (**lines 423-431**)

In response to the reviewer's request to tone down the Abstract, Methods, Results, and Discussion, we have made appropriate revisions throughout the manuscript. We have emphasized that the effect direction is consistent but modest in size and noted the heterogeneity across datasets. Additionally, we discuss potential sources of this heterogeneity. (**lines 71-75; 634-638**)

**Comments to the Author**

2.Reproducibility basics. Include a compact SNP table (allele, beta/SE, freq, per-SNP F), spell out clumping/harmonization rules, state the multiple-testing plan (FDR vs Bonferroni) with "significant" vs "suggestive" labels, and link code/data so the pipeline is rerunnable.

**Author response and action taken:**

We thank the reviewer for raising this important point on reproducibility. We have taken the following steps to address the concerns:

1. Compact SNP Table:We have included a compact SNP table that contains the following columns: allele, beta/SE, frequency, and per-SNP F-values, which are essential for reproducibility. This table summarizes the SNPs used in our Mendelian randomization analysis, and we have added it as **Supplementary Table 11**.

Clumping/Harmonization Rules:We have explicitly spelled out our clumping and harmonization rules for SNP selection and alignment. Specifically, we performed clumping to ensure SNP independence (r² < 0.001, 10,000 kb window). Harmonization ensured consistent effect alleles across datasets by comparing effect directions and allele frequencies, excluding inconsistent SNPs. These steps were implemented using the "TwoSampleMR" package in R. (**lines 207-211**)

3. Multiple-testing Plan:We followed a multiple-testing correction strategy using the FDR for all Mendelian randomization analyses, as described in the Methods section. We have clearly marked the "significant" results with FDR-corrected p-values < 0.05, and we have labeled the "suggestive" results as those with p-values between 0.05 and 0.1.
4. Code and Data Availability: To ensure research transparency, all datasets (CTSH, lung cancer GWAS, and metabolic trait data) are sourced from public repositories (IEU OpenGWAS, GWAS Catalog, FinnGen), with links provided in the “Availability of data and materials” section. Analysis code (including R code) is available upon request from the corresponding author. We are committed to supporting research reproducibility.

**Comments to the Author**

3.Single-cell minimum viable package.

Show one UMAP with CTSH violins and report the key macrophage contrast (logFC + FDR).

Add two lines clarifying the public expression resources you used (e.g., TCGA/HPA dataset names, sample counts, processing settings) and how the tumor vs normal groups were defined.

**Author response and action taken:**

Thank you for your valuable suggestions on single-cell analysis and public expression resources. We have made the following revisions:

**Single-Cell Analysis:** We extracted mo-Mac (n = 5,452) and Alveolar Mac (n = 11,824) subtypes from the GSE131907 dataset (n = 89,902 cells), created a new Seurat object (v5.1.0), and generated an integrated figure (**Supplementary Figure 13**). The left UMAP shows cell distribution (based on PCA dims 1:10, RunUMAP), and the right violin plot shows CTSH expression (log2(TPM+1)). The mo-Mac vs Alveolar Mac contrast yields logFC = 0.319, FDR = 5.059504e-09 (p = 1.707331e-13, two-sided Wilcoxon rank-sum test, p < 0.0001, **** indicates significance). The figure legend has been updated to include sample sizes, statistical methods, and significance symbols. (**lines 512-519**)

**Public Expression Resources:** In the methods, we added: TCGA-LUAD (n = 477 tumor samples, 59 normal samples, log2-normalized RSEM processing) was used for CTSH differential expression and survival analysis, with tumor samples defined as confirmed lung adenocarcinoma and normal samples as adjacent tissues; HPA (immunohistochemistry images, n = 10 tumor/normal sample pairs) was used for protein expression validation, with tumors defined by pathological diagnosis. Processing settings include log1p normalization for GSE131907 (Seurat v5.1.0) and RSEM normalization for TCGA. (**lines 169-174**)

**Revision with responses: 02-Nov-2025 ( 3^rd^ Revision )**

In this version, the previously used images Figure 6N has been replaced with the original gel blots, and the sections on Statistical Analysis and Figure Legends have been further refined and supplemented.
